# Creating a Nutrient-Dense Menu Using Foods Consumed by Native Communities in the Northern Great Plains Prior to 1851 for Use in Dietary Intervention Trial

**DOI:** 10.1016/j.cdnut.2025.107522

**Published:** 2025-08-05

**Authors:** Julie M Hess, Jacob D Bourboun, Madeline E Comeau, Brooke Froelich, Debra Fossum, Angela Scheett, Edwin Kitzes, Tiffany Baker-Ramsey, Dale C Brunelle, James N Roemmich

**Affiliations:** 1 USDA-ARS Grand Forks Human Nutrition Research Center, Grand Forks, ND, United States; 2 University of North Dakota, Grand Forks, ND, United States; 3 United Tribes Technical College, Bismarck, ND, United States; 4 Sitting Bull College, Fort Yates, ND, United States

**Keywords:** indigenous, healthy diet, historical diet, food policy, dietary guidance, native foods, Great Plains

## Abstract

**Background:**

Indigenous communities experience greater rates of health disparities than surrounding areas, in part due to a forced transition away from traditional diets.

**Objectives:**

The purpose of this study was to develop a dietary intervention (menu) based on traditional foods consumed by indigenous peoples who lived in the Northern Great Plains region near modern-day Grand Forks, North Dakota (ND), prior to the year 1851.

**Methods:**

Three key factors informed this menu—*1*) nutrient composition; *2*) traditional foods; and *3*) practicality for contemporary consumers using modern preparation methods. Food pattern modeling was applied to the Healthy United States–Style Dietary Pattern from the 2020 Dietary Guidelines for Americans to determine the nutrient composition of a 2400 kcal eating pattern, used as the goal nutrient composition. To define “traditional” foods, a list of foods and beverages consumed by indigenous peoples in the Grand Forks, ND, area prior to 1851 (when the Indian Appropriations Act established reservations) was developed through historical research. Recipes were refined and tasted for the seasoning level by trained kitchen staff in collaboration with an indigenous chef and leader.

**Results:**

On average, the 5-d menu provided adequate nutrition, including sufficient fiber (50.5 g), potassium (4606 mg), and iron (22.5 mg). Sodium (2828 mg) exceeded the 2300 mg recommendation, whereas saturated fat (6.5% of total calories) was provided within recommendations. The menu fell below recommendations for calcium (617 mg) and vitamin D (4.2 μg).

**Conclusions:**

A menu comprising foods traditional to indigenous communities of the Northern Great Plains meets some contemporary and evidence-based recommendations for healthy eating patterns.

## Introduction

Forced movement from ancestral lands to reservations, the decimation of buffalo, and an array of other factors in the mid-19th century led to dramatic changes in diet and lifestyle among the Great Plains Indians. Hunger was a persistent problem on reservations, where American Indians relied on the government to distribute rations, which were inconsistent and insufficient [1]. These involuntary lifestyle changes likely contributed to higher rates of health problems among this population that persist today, including diabetes, heart disease, and obesity [2,3]. Hunger, now more commonly framed as “food insecurity,” or access to enough food at all times for an active, healthy life [4], remains a contemporary concern among American Indians and Alaska Natives [5]. In the state of North Dakota (ND), food insecurity rates remain disproportionately high among tribal communities [6]. On tribal reservations in ND (Fort Berthold, Spirit Lake, Standing Rock, and Turtle Mountain), average food insecurity rates affect 5%, 11%, 15%, and 16%, respectively, of the population [6]. In contrast, food insecurity in the state overall affects ∼9% of residents [7]. The United States Department of Agriculture’s (USDA’s) Agricultural Research Service Grand Forks Human Nutrition Research Center (GFHNRC) in Grand Forks, ND, is located within close geographic proximity to several federally recognized tribal communities. Not only do some of these communities experience greater food insecurity rates due to the forced transition away from traditional diets but also they do have greater rates of health disparities than populations in surrounding nontribal areas [8]. Food security is often presented as a component of food sovereignty, a broader concept that encompasses the right of communities to manage their food production, ecological resources, and markets [9,10]. Indigenous food sovereignty is fundamentally distinct from food security, because the economic focus of food security is rooted in Euro-American food systems [11]. Supporting and promoting traditional foods is integral to indigenous food sovereignty [12]. Traditional foods utilize native plants and animals that are indigenous to the area where a native tribe resides [13]. Dr. Tara Maudrie, a graduate of the Johns Hopkins Center for Indigenous Health, builds on this definition describing traditional foods as “foods that come from the land, have sustained our ancestors since time immemorial, are part of our spiritual relationships, have seasonal variance, and are nutrient-dense” [14]. She contrasts traditional foods with “cultural foods,” which she defines as those that “arise from a time of hardship, enforced on Native peoples, through commodities, calorically dense, may carry emotion or meaning but not spiritual significance” [14], providing bison as an example of traditional food and fry bread as an example of cultural food.

To work toward greater support and promotion of traditional foods for tribal communities in and around ND, the GFHNRC planned an intervention study to assess the impact of consuming a diet rich in traditional foods on the coactivation of physical activity among indigenous peoples living in the greater Grand Forks community (clinicaltrials.gov identifier: NCT06674642). This manuscript describes the development of the dietary intervention (menu) used in the intervention study mentioned above. This menu is based on traditional foods that were part of the food systems of indigenous peoples who lived in the Northern Great Plains prior to the year 1851, when reservations were established in the region. Throughout this article, the term “indigenous” refers to native peoples and communities living in the Northern Great Plains region prior to 1851. More detail is provided about these communities in the section below, titled Tribes of the Great Northern Plains.

## Methods

The menu was developed in partnership with representatives from the United Tribes Technical College in Bismarck, ND, and the University of North Dakota (UND) to meet 3 primary objectives:

1. Align with nutrient recommendations from the 2020–2025 Dietary Guidelines for Americans (DGA) [15];
2. Contain 80% energy from traditional foods that would have been consumed in the Northern Great Plains prior to the establishment of reservations in 1851; and
3. Be possible to prepare in a commercial kitchen and package for participants to reheat and eat at home as part of a controlled feeding study.

The aim of this study was to identify traditional foods that could be prepared with modern cooking techniques, adapted for modern palates, and used as a healthy dietary menu as part of a human intervention study.

### Nutrient content and the DGA

Because indigenous communities do not traditionally consume dairy foods, which are not native to the Americans [16], the Healthy Eating Index (HEI) was not appropriate for assessing the diet quality of a traditional indigenous menu. In lieu of the HEI, the nutrient composition of the Healthy United States–Style Dietary Pattern (HUSDP) from the 2020 DGA was used to assess nutrient adequacy and ascertain how a menu comprising traditional foods mapped onto contemporary standards of nutrition science.

To determine the nutrient composition, the 2020 Food Pattern Modeling Report [17] was used to calculate the concentration of nutrients in the 2400 kcal HUSDP by multiplying the nutrients provided in each food group composite by the number of cup-equivalents or ounce-equivalents of each food group in the 2400 kcal HUSDP [15]. The nutrient content of the menu was determined using the USDA National Nutrient Database for Standard Reference, Release 27 [18], through use of a customized in-house nutrient analysis program at the GFHNRC. Each menu item was individually matched with its equivalent in the proprietary analysis program via close evaluation of ingredients, energy, and nutrient content by trained research staff. Certain items were not reflected in this database, including aronia berry juice, purple potato, Dash seasoning, Dijon mustard, and venison sausage. For these items, the closest substitutes were identified in the nutrient analysis program.

### Identifying traditional foods

#### Tribes of the Great Northern Plains

Given the importance of locality to indigenous foods, foods that would have been consumed by indigenous peoples who lived in the general geographic area near Grand Forks, ND, were selected. Because the GFHNRC is adjacent to UND’s campus, the university’s Land Acknowledgment Statement [19] from 2020 (below) was used to draft an initial list of tribes to include in historical food research. This statement reads as follows:

*Today, the University of North Dakota rests on the ancestral lands of the Pembina and Red Lake Bands of Ojibwe and the Dakota Oyate – presently existing as composite parts of the Red Lake, Turtle Mountain, White Earth Bands, and the Dakota Tribes of Minnesota and North Dakota. We acknowledge the people who resided here for generations and recognize that the spirit of the Ojibwe and Oyate people permeates this land. As a university community, we will continue to build upon our relations with the First Nations of the State of North Dakota – the Mandan, Hidatsa, and Arikara Nation, Sisseton-Wahpeton Oyate Nation, Spirit Lake Nation, Standing Rock Sioux Tribe, and Turtle Mountain Band of Chippewa Indians.*

Broadly, the groups whose foodways were utilized as the basis for this menu include the Lakota; the Dakota or Santee; the Western Dakota; Plains Ojibwa; and Mandan, Hidatsa, and Arikara Nation. A more detailed list is provided in Table 1 [[20], [21], [22]].

**TABLE 1:** Tribal communities whose foodways informed the healthy indigenous menu.

Oceti Ŝakowiŋ or Oceti Sakowin—the Seven Council Fires of the Great Sioux Nation [20]	
Dakota or Santee	Mdewakanton Wahpekute Wahpeton Sisseton
Western Dakota or Nakota	Yankton Yanktonai
Lakota or Teton	Hunkpapa Brulé Oglala Sans Arc Miniconjou Blackfeet Two Kettles [21]
Plains Ojibwa	Turtle Mountain Band of Chippewa • Pembina Band of Chippewa Indians • Métis
MHA Nation [22]	Mandan Hidatsa Sahnish—Arikara

#### Timeline

The year 1851 was chosen because it marked the beginning of a transitional period for the indigenous peoples living in Minnesota and on the Great Plains. This period was marked by a series of treaties with the federal government that altered the lifestyles of indigenous peoples, including their diets. Marked by the passing of the Indian Appropriation Act, 1851 saw the creation of the reservation system in the United States. The era during which reservations were established (1851–1878) began on February 27, 1851, when Congress passed the Indian Appropriation Act, also known as the Appropriation Bill for Indian Affairs [23]. This act authorized the establishment of reservations in what is now Oklahoma and codified the means of creating, funding, and administrating reservations into law. The reservations modeled after this act had a significant impact on diets because they restricted the ability of indigenous people to hunt and gather food. Instead, it compelled those living on reservations to utilize the food subsidies, which were negotiated in their treaties for survival. This dependency on food supplied by the federal government led to the introduction of nontraditional foods and the replacement of traditional foods in indigenous diets.

In the summer of 1851, the federal government signed treaties with the Dakota people living in the Minnesota Territory. Collectively known as the 1851 Dakota Land Cession Treaties, the Treaties of Traverse des Sioux (July 23, 1851) and Mendota (August 5, 1851) purchased millions of acres of land in exchange for a large monetary sum distributed to the Dakota people annually as annuity payments. Additionally, these treaties also set aside large tracts of land to become the "home of Dakota Indians" and stipulated that part of the annuities would be used for "general agricultural and improvement of civilization fund" and "[f]or the purchase of goods and provisions" [24]. These goods, usually purchased from unscrupulous traders operating on the reservations, included flour, pork, beef, and other nontraditional foodstuffs. This dependency on traders to supply them with food, especially in the winter months, forever altered the way of life of the Dakota people [25].

These conditions came to a head in 1862, when large scale violence between the United States and Dakota warriors broke out across the Minnesota River Valley. The United States–Dakota War of 1862 was the largest ethnic conflict in the American history and would have far-reaching consequences [26]. Among the many injustices perpetrated on the Dakota people in the aftermath of the war was the abrogation of all treaties and their exile from Minnesota. The Dakota Diaspora forced thousands of Dakotas to relocate to reservations far away from their traditional homelands. Two of these reservations, the Fort Totten Indian Reservation (Spirit Lake) and the Lake Traverse Reservation (Sisseton–Wahpeton Oyate), were established in the Dakota Territory. Living on these reservations and away from their homelands, the Dakota had to adapt their diets to their new environment and conditions.

The Horse Creek Treaty—the Treaty of Fort Laramie 1851—represented a pivotal moment for the indigenous tribes living on the Great Plains [27]. Written for the "purpose of establishing and confirming peaceful relations" among the Great Plains tribes, the Horse Creek Treaty recognized the tribal territories and boundaries [28]. Although it did not have as immediate an impact as the Dakota Land Cession Treaties, it did start the process of defining the territories in which the tribes could live and hunt. Furthermore, it included provisions guaranteeing the federal government would "deliver to said Indian nations the sum of fifty thousand dollars per annum for the term of fifty years" if these nations upheld the terms of the treaty [24]. These payments, however, were to be delivered in "provisions, merchandise, domestic animals, and agricultural implements" [24]. The treaty did not have such an immediate impact on the Sioux, who would not enter the reservation era until the 1870s [29]. For others, like the Mandan, Hidatsa, and Arikara (Sahnish), it represented a dramatic shift in their way of life. It would bring 3 culturally similar but distinct tribes together on a territory that did not include the Mandan and Hidatsa settlement of Like-a-Fishhook Village [30]. Furthermore, the Mandan, Hidatsa, and Sahnish people, who had all been devastated by the 1837 Smallpox Epidemic, had become reliant on government provisions for survival. The Fort Laramie Treaty and the decline of the buffalo trade furthered their reliance on provisions and further introduced nontraditional foods into their diet. These treaties, specific to communities in what is now ND and Minnesota, created reservation systems, provided for allotments, formed nations of disparate tribes, and forever altered the signees’ way of life, including their food selection, choices, and preparation.

#### Selection of foods to using historical documents

Table 2 [[31], [32], [33], [34], [35], [36], [37], [38], [39]] identifies which foods included in the healthy indigenous diet were utilized by each tribe, according to primary and secondary sources. The selection of foods to include adopted an ethnohistorical approach, accepting and incorporating multiple epistemologies, although privileging indigenous sources. Unlike traditional histories that focus on the written word, indigenous societies preserve their past through oral records [40]. There is little difference between storytellers and historians in societies like the Arikara [30]. Through stories, indigenous societies preserve their history, traditions, spiritual understandings, and identity [41]. Moreover, food and its consumption are prominent throughout indigenous oral traditions, because they are intimately connected to spirituality, traditions, and way of life. Therefore, the first sources utilized to determine what food was regularly consumed by each tribe were oral accounts. Other indigenous sources include winter accounts, syllabary, and petroglyphs [40].

**TABLE 2:** Tribal origin and References for Primary Ingredients in Healthy Indigenous Menu.

Hidatsa [[31], [32], [33]]	Ojibwe [34]	Chippewa [[35], [36], [37]]	Arikara [33,38]	Mandan [33,39]	Sioux [33,38]
Beans	Blackberry	Blackberry	Beans	Beans	Berries
Buffalo	Blueberry	Blueberry	Buffalo	Buffalo	Buffalo
Corn	Buffalo	Buffalo	Corn	Corn	Chokecherries
Cornmeal	Cabbage	Chokecherry	Squash	Grains	Corn
Hominy	Cedar	Corn		Oats	Maple sugar
Salt	Chokecherry	Cranberry		Peas	Nuts
Squash	Cranberries	Duck^1^		Pumpkins^1^	Peas
Sugar^2^	Eggs^3^	Ginger^1^			Plums
Sunflower seeds	Grapes	Grape (raisins)			Prairie turnip
	Juniper	Hazelnut			Roots
	Onions	Juneberry			Wild rice
	Red pepper	Maple (syrup)			
	Strawberry	Mountain mint/wintergreen			
	Trout	Pumpkin^1^			
	Walnuts	Red raspberry			
	Wild greens	Squash			
	Wild rice	Strawberry			
		Wild cherry^1^			
		Wild rice			

1 Not included on final menu but listed in sources.

2 Source did not specify what type of sugar.

3 Turkey eggs, gull eggs, and fish eggs specifically are noted as food sources in John Tanner’s account.

There are, however, a few methodological disadvantages within a Western/Eurocentric biomedical cultural perspective to using native oral histories. Oral histories can be problematic sources within the Euro-American cultural framework due to different preferences for knowledge transmission [42]. Oral histories of native communities can also be challenging to access because of historical disruptions in the integrity and cohesion of oral traditions through land dispossession [43] and the sacredness of these histories to individual tribes. Protecting the integrity of oral histories means that very few outsiders have had the privilege to hear them and even less opportunity to record them for posterity [30].

A select outsiders have been allowed to record some stories. Sources like *Waheenee: An Indian Girl's Story Told by Herself to Gilbert L. Wilson* and *Buffalo Bird Woman's Garden: Agriculture of Hidatsa Indians* provided valuable information for this study [31]. Both books were produced from interviews with an elderly Hidatsa woman named Waheenee or Buffalo Bird Woman. They gave a detailed account of Hidatsa life, culture, and traditions before 1851. These sources also provide detailed accounts of the traditional Hidatsa diets and the types of food consumed. Given the linguistic and cultural similarities and the close geographical proximity of the Hidatsa and Mandan, Waheenee's accounts give us a better understanding of both tribes. Published oral traditions relating to the other indigenous peoples were also consulted [[44], [45], [46]].

This study did not rely exclusively on indigenous sources. Applying the historical method, journals, memoirs, and correspondence, particularly those of fur traders and missionaries, were the most common sources used alongside military logs, government reports, and Congressional documents. Other sources, like the painter George Catlin's *Manners, Customs, and Conditions of the North American Indians*, gave a detailed description of indigenous ways of life, ceremonies, and food systems and provided excellent material culture through paintings of influential tribal leaders and landscapes [47]. Catlin’s book discusses most tribes—the Plains Ojibwa, Sioux, the Mandan, Hidatsa, and Arikara. These “outsider” accounts of 19th-century indigenous life provide insight into traditional foods.

Some sources by nonindigenous writers provided derogatory and dismissive perspectives of indigenous ways of life and culture and had to be viewed critically. For example, Edwin Thompson Dening, a fur trader who lived with Arikara in the 1830s, wrote that the "domestic character and habits of the Arickaras [sic] are decidedly more filthy than any other nation…In their dress they are greasy and slovenly" [38]. Dening does give us a detailed account of Sahnish diets and how they prepared and cooked their food. Even when being "complimentary," however, some of these authors managed to be insulting. Catlin, who by all accounts was impressed with the Mandan, regularly referred to them as "savages" and found many of their customs "uncivilized."

John Tanner’s narrative of his capture and life among the Ojibwe was a valuable source [34]. After a brief captivity with Odawa, Tanner comes to live with the Saulteaux or Plains Ojibwe in the Red River and Great Lakes region [48]. Because starvation was a constant throughout his travels, Tanner’s accounts detail the many different types of food he and those around him consumed. From hunting large game and fowl to gathering berries and making sugar, his narrative provides a comprehensive, and with the inclusion of an index discussing everything from language to music, an encyclopedic account of the Plains Ojibwa life, culture, and food systems [34].

Tanner’s narrative also shows the interconnectedness of food systems among people across large geographical spaces. In his travels, he regularly encountered many different indigenous peoples and tribes. Whether meeting in battle, at trading posts, or as part of a hunting party, each interaction represented a cross-cultural exchange. Food played a central role in these interactions. Tanner also regularly visited Pembina, where a sizable population of Métis resided. Their deep biological and cultural connections with the Ojibwa and the white fur traders meant they also developed a unique food system. Regular interactions with the Métis at Pembina and other places along the Red River meant that Tanner and his Ojibwa companions consumed foods like pemican (a mixture of animal fat, dried meat, and dried berries). These kinds of interactions added to the diversity and interconnectedness of food systems throughout the Great Plains and Great Lakes region.

All tribes included (Hidatsa, Ojibwe, Chippewa, Arikara, Mandan, and Sioux) consumed some foods in common, notably buffalo. Although there may be more overlap among the foods regularly consumed by different tribes, historical records do not contain a detailed list of individual foods consumed by each people; therefore, knowledge of the dietary patterns of these communities remains limited.

#### Obtaining and preparing foods for intervention study menu

To ensure that this menu would contain primarily traditional foods but would remain palatable for modern consumers and accessible from a contemporary grocery store, the research team aligned on the parameters below for the menu:

1. Up to 20% of energy on the menu could come from foods not historically consumed by indigenous communities in the Northern Great Plains.
2. Seasonings, including salt, would be added to match the palate of modern consumers. The appropriate seasoning level would be determined via taste testing procedures used for all intervention menu developments.
3. Ingredients would be purchased from a local Midwestern grocery store chain or a specialty butcher store. Certain shelf-stable specialty ingredients, such as powdered aronia berries, would be purchased from Amazon.com.

The process of defining the practicality of food preparation and suitability for modern consumers, appliances, and palates was an iterative process, involving metabolic kitchen staff, research dietitians, tribal representatives, and a team of research nutritionists. Certain choices, such as the choice to include ground meats and salt in this menu, were made to ensure this menu would contain items easier to prepare and reheat and to ensure palatability for a modern consumer, although the use of these products is a departure from historical accuracy.

## Results

### Reverse engineering of DGA diet

Table 3 lists the nutrient composition of the 2400 kcal HUSDP, which was used as a guide for the development of a nutrient-dense healthy indigenous dietary pattern. The HUSDP contains slightly less fiber (90% of recommendations), vitamin E (75%), vitamin D (56%), choline (75%), and selenium (34%) than dietary reference intakes for males aged 19–30 y for whom a 2400 kcal diet may be appropriate. As noted in Table 3, the HUSDP meets or exceeds nutrient recommendations for all other micronutrients and macronutrients assessed.

**TABLE 3:** Nutrient composition of 2400 kcal Healthy United States–Style Dietary Pattern (HUSDP) from the 2020–2025 Dietary Guidelines for Americans.

Nutrient	Amount in HUSDP	Recommended dietary reference intakes (DRI)	% DRI
Calories (kcal)	2405	2400	100
Protein (g)	108	56	193
Protein (% kcal)	18	10–35	Within range
Carbohydrate (g)	276	130	212
Carbohydrate (% kcal)	46	45–65	Within range
Fiber (g)	30.6	34	90
Total fat (g)	66.2	n/a	n/a
Fat (% kcal)	25	20–35	Within range
Saturated fat (g)	13	n/a	n/a
Saturated fat (% kcal)	5	<10	Within range
Monounsaturated fat (g)	23.5	n/a	n/a
Polyunsaturated fat (g)	23.9	n/a	n/a
Linoleic acid (g)	21	17	124
Linolenic acid (g)	2.6	1.6	162
Cholesterol (mg)	245	As low as possible	n/a
Minerals			
Calcium (mg)	1367	1000	137
Iron (mg)	18	8	225
Magnesium (mg)	423	400	106
Phosphorus (mg)	1899	700	271
Potassium (mg)	3849	3400	113
Sodium (mg)	1907	2300	83
Zinc (mg)	15	11	136
Copper (mg)	2	0.9	222
Selenium (μg)	137	400	34
Vitamins			
Vitamin A, RAE (μg)	981	900	109
Vitamin E, α-tocopherols (mg)	11	15	75
Vitamin D (International Units)	338	600	56
Vitamin C (mg)	142	90	158
Thiamin (mg)	2.2	1.2	183
Riboflavin (mg)	2.2	1.3	169
Niacin (mg)	28	16	175
Vitamin B_6_ (mg)	2.5	1.3	192
Vitamin B_12_ (μg)	7.0	2.4	292
Choline (mg)	415	550	75
Vitamin K (μg)	173	120	144
Folate, dietary folate equivalents (μg)	651	400	163

### Foods with documented historical use pre-1851

Table 2 lists the food items found in different historical sources by tribe. For instance, the Buffalo Bird Woman’s Garden source [31] mentions that cornmeal, squash, beans, corn, and hominy were consumed by the Hidatsa, and an 1877 account about the Hidatsa [32] notes that salt, bison, sunflower seeds, and sugar were consumed by this group.

Additional items added to the menu that were not found in historical sources included primarily items used for seasoning and flavor, including celery seeds, cilantro, cinnamon, cumin, mustard, lemon/limes, parsley, and vinegar. Chia seeds were also used. Although some of these foods may be native to the United States and may have been used in the diets of indigenous peoples approximated in this work, there is no evidence to support that they were available. These items comprise 24% of the energy in the menu (see Supplemental Files for a full list of nontraditional foods included and their energy contributions to the menu). Purple potatoes and the use of maple syrup or honey instead of cane or beet sugar were included based on recommendations from an indigenous chef.

### Menu

The nutrient content of the final menu can be found in Table 4, and the healthy indigenous menu itself can be found in Table 5. The original sources of recipes can be found in Supplemental Table 1, and the list of ingredients included within final recipes on the menu (e.g., cornmeal mush, manoomin meatballs, 3 sisters stew, etc.) can be found in Supplemental Tables 2–40. On average, the 5-d menu (Table 4) provided adequate fiber (41.5 g), potassium (4556.5 mg), and iron (22.6 mg) and provided macronutrients (protein, carbohydrate, and fat) within acceptable macronutrient distribution ranges (AMDRs). Sodium (2668 mg) exceeded the 2300 mg recommendation, whereas saturated fat (6.5% total calories) was provided within recommendations. The menu fell below recommendations for calcium (586.2 mg), vitamin A (694.2 μg), and vitamin D (414 International Units). Photos of all prepared meals can be found in Supplemental Figures 1–15.

**TABLE 4:** Nutrition composition of 2400 Healthy Indigenous Dietary Pattern.

Nutrients	Source of goal^1^	2400 kcal Healthy indigenous menu	Nutritional goals
		(daily average)	(Male 19–30 y)^1^
Energy (kcal)		2389.5	2400
Macronutrients			
Protein (g)	RDA	124.5	56
Protein (%en)	AMDR	20.3	10–35
Carbohydrate (g)	RDA	306	130
Carbohydrate (%en)	AMDR	49.9	45–65
Fiber (g)	DGA	41.5	34 (14 g/1000 kcal)
Added sugars (%en)	DGA	n/a	<10
Total lipid (g)	n/a	81.2	n/a
Total lipid (%en)	AMDR	29.8	20–35
Saturated fat (g)	n/a	17.8	n/a
Saturated fat (%en)	DGA	6.5	<10
18:2 Linoleic acid (g)	AI	16.1	17
18:3 Linolenic acid (g)	AI	3	1.6
Minerals			
Calcium (mg)	RDA	586.2	1000
Iron (mg)	RDA	22.6	8
Magnesium (mg)	RDA	560.9	400
Phosphorus (mg)	RDA	2000	700
Potassium (mg)	AI	4556.5	3400
Sodium (mg)	CDRR	2668	2300
Zinc (mg)	RDA	20.6	11
Vitamins			
Vitamin A (μg RAE)	RDA	694.2	900
Vitamin E, α-tocopherols (mg)	RDA	17.9	15
Vitamin D (International Units)	RDA	414	600
Vitamin C (mg)	RDA	191.8	90
Thiamin (mg)	RDA	1.6	1.2
Riboflavin (mg)	RDA	2.6	1.3
Niacin (mg)	RDA	31.2	16
Vitamin B-6 (mg)	RDA	3.1	1.3
Vitamin B-12 (μg)	RDA	7.8	2.4
Choline (mg)	AI	571.6	550
Vitamin K (μg)	AI	221.9	120
Folate, dietary folate equivalents (μg)	RDA	668.7	400

AI, adequate intake; AMDR, acceptable macronutrient distribution range; CDRR, chronic disease risk reduction; DGA, Dietary Guidelines for Americans; RDA, Recommended Dietary Allowance.

1 Used as reference for 2400 kcal dietary pattern based on 2020–2025 Dietary Guidelines for Americans Table A1–2.

**TABLE 5:** A 2400 kcal healthy indigenous menu.

	Breakfast	Lunch	Supper
Day 1	• Cornmeal Mush (260 g) served with raisins (50 g), maple syrup (40 g) and walnuts (20 g) • Hard-cooked egg (100 g)	• Aronia Berry (chokeberry) Drink made with water (240 g) mixed with aronia powder (10 g) and 20 g maple syrup • 3 Sisters (corn, beans, squash) stew (360 g) with venison tips (30 g) and ground bison (50 g) • Mixed Greens (60 g) topped with raisins (40 g), walnuts (15 g), and vinaigrette dressing (25 g) • Wild Rice Flatbread (90 g) (cooked wild rice mixed with egg, chia, and seasoning and formed into flatbread)	• Salmon (160 g) topped with 10 g lemon juice and 2 g lemon pepper served on a bed of wild rice (200 g) • Steamed green beans (180 g) seasoned with sunflower oil (1 g) and ground juniper (0.3 g) and topped with walnuts (15 g) • Berry Fruit Salad made with 200 g mixed berries topped with a dressing made with honey (20 g) and lime juice (10 g)
Day 2	• Eggs (100 g) scrambled with sweet potato (90 g), green bell pepper (50 g), green onion (10 g) • Cooked amaranth (300 g; a whole grain porridge) served with a mixed berry sauce called wojape (200 g) and maple syrup (30 g)	• Masa corn cakes (corn tortillas; 78 g) served with seasoned ground bison (165 g) and mashed pinto beans (180 g) • Roasted vegetables including green bell peppers (50 g), red bell peppers (50 g), and yellow onion (60 g) • Radish salsa (60 g) including radishes, garlic, jalapeno, lemon juice, cilantro, seasoning	• Bison Hash (300 g) made with ground bison, onion, sweet potato, turnip, squash, and wild rice • Corn muffin (55 g) served with honey (25 g) • Sunflower cookie (60 g) made with sunflower butter, maple syrup, and cornmeal
Day 3	• Wild rice porridge (360 g; wild rice cooked with hazelnut milk) and served with wojape (240 g; a mixed berry sauce cooked with 45 g honey) and served with honey (20 g) • Venison Sausage (240 g)	• Salmon patty (184 g) made with canned salmon, egg, lemon, mustard, dill, and masa flour served with lemon dill sauce made with avocado oil-based mayonnaise (27.5), dill weed (0.2 g), and lemon juice (2.5 g) • Pinto Beans (320 g) braised with onion and garlic • Summer Squash Salad made with zucchini (100 g), yellow squash (100 g), parsley (10 g), and green onion (15 g) served with a vinaigrette dressing of sunflower oil (9 g), cider vinegar (10 g), salt (2 g), and ground juniper (0.3 g)	• Elk burgers (186 g) • Mixed Green Salad (70 g) served with black beans (120 g) and vinaigrette dressing (30 g) • Stewed Zucchini (300 g) made with onions, tomatoes, and wild rice • Wild Rice Flatbread (120 g)
Day 4	• Eggs (150 g) scrambled with mushrooms (30 g), onion (30 g), and spinach (40 g) • Purple potato (270) roasted with seasoning (2 g) and sunflower oil (6 g) • Dried apricots (65 g)	• Bison chili (280 g) made with ground bison, onion, pinto beans, tomato, and seasonings • No mayo coleslaw made with cabbage (120 g), green onion (10 g), apple cider vinegar (25 g), maple syrup (15 g), mustard (15 g), celery seed (0.5 g) • Corn muffin (110 g) served with honey (50 g)	• Aronia Berry (chokeberry) Drink made with water (240 g) mixed with aronia powder (10 g) and 20 g maple syrup • Walleye (220 g) seared in sunflower oil (5 g) and seasoned with salt (0.8 g) and pepper (0.5 g) • 3 Sisters salad made with black beans (110 g), corn (90 g), zucchini (100 g), yellow squash (100 g), and onion (10 g) served with a dressing of maple syrup (10 g), cider vinegar (10 g), and sunflower oil (10 g) • Spaghetti squash (115 g) mixed with pesto (25 g)
Day 5	• Potawatomi berry rice made of wild rice (160 g) and mixed berries (80 g) combined with maple syrup (10 g) and ground cinnamon (0.8 g) • Walnuts (15 g) • Hard-cooked egg (100 g)	• Manoomin (wild rice) Elk Meatballs (224 g) made of elk mixed with wild rice, egg, onion, and seasoning • Polenta (200 g; cornmeal porridge made with water and salt) • Succotash (220 g) made with corn, lima beans, red bell pepper, tomatoes, onion, and seasoning	• Roast turkey (160 g) • Gooseberry compote (60 g), a mixture of canned gooseberries cooked with dried cranberries • 3 Sisters mash made with braised beans (270 g), a combination of onion, zucchini, braised pinto beans, maple syrup, hominy, and seasoning • Wild rice flatbread (90 g) • Caramelized seed mix (40 g) made with pepitas, sunflower seeds, and walnuts coated in melted maple sugar

## Discussion

The final traditional indigenous menu is nutrient dense and provides adequate amounts of some nutrients of concern for the United States population, including potassium, iron, and fiber [15]. The menu also remained within recommendations for saturated fat content (6.5% of total energy) and within AMDRs for all macronutrients (protein = 20.3% energy; carbohydrate = 49.9% energy; and fat = 29.8% energy). The menu also provided adequate amounts of most micronutrients. However, the menu did not contain adequate amounts of 2 nutrients of concern—calcium and vitamin D, likely due to the absence of dairy foods or fortified soy alternatives on this menu.

In creating this menu, the goal was to include 80% of energy from traditionally indigenous foods from the Great Plains region. The final menu included 76% of energy from traditional foods. However, some of the foods identified as nontraditional (garlic, lemons, cilantro, parsley, and others listed in Supplemental Table 41) may have been included in pre-1851 dietary patterns and were simply not recorded, especially because many of these food items are flavorings added in small amounts.

Traditional ingredients noted in historical records were used as the base of the study menu. Yet there were several constraints to the historical accuracy of the final menu for reasons ranging from practicality to safety to lack of information. Some substitutions were used in lieu of the original foods mentioned in historical accounts. For instance, in his account of captivity with the Ojibwe people [34], John Tanner described the consumption of turkey eggs, gull’s eggs, and fish eggs. However, the traditional indigenous menu uses chicken eggs, as these are more widely used, accepted, and accessible. Some foods reported in historical accounts are no longer widely consumed or available for purchase as foods. For instance, dog meat, beaver tail, and bison tongue are described as delicacies [47] and treasured feast items consumed by some tribes prior to 1851. These items were not included in this menu as they are no longer widely consumed. To ensure that this menu would be acceptable to modern indigenous communities, an indigenous chef and a representative from United Tribes Technical College in Bismarck, ND, tasted every menu item prepared by the GFHNRC kitchen staff and provided input and feedback on how to tailor the foods and beverages provided.

Aronia berry, or “chokeberry,” juice is included on days 1 and 4 of the final study menu, and this recipe was made using black chokeberries. The indigenous peoples in North America consumed many different fruits and berries, including chokecherry (*Prunus virginiana*) and black chokeberry (*Aronia melanocarpa*) [49,50]. Chokecherry was widely distributed throughout the Northern Great Plains, whereas black chokeberry was distributed near the eastern boundary of the Northern Great Plains near the Great Lakes and further east [51]. These berries were eaten fresh, dried, boiled, or mixed with meat to produce pemmican [50,52]. Black chokeberry was added to the menu because it is commercially available as frozen fruit, concentrated extract juice, or dried powder, whereas we could find chokecherry only available as a frozen fruit or in already processed foods, such as syrups and jams. Also, the pit of the chokecherry contains cyanogenic glycoside prunasin [53], which raises safety issues or requires removal that is labor intensive. In addition, there is ample research on the effects of black chokeberry, revealing its antioxidant [54] and anti-inflammatory properties [55]. Although we have not found any accounts of black chokeberry being made into a juice by indigenous peoples of the Northern Great Plains, our menu includes it as a juice. Black chokeberry juice presents a distinct traditional and culturally significant food in a modern way in which we hope aids in connecting the indigenous peoples of the Northern Great Plains with their traditional foods.

It is also not known whether the foods used in the menu would have been consumed in the same way that they are now. For instance, although some sources mention onion as an indigenous food, it is not known if it would have been used before 1851 to flavor foods in the same manner as it is today. All food preparation used for this menu is a departure from traditional methods. Many foods used in our menu would have been foraged and would have looked and tasted quite differently from the modern equivalents. As one example, prairie turnips were frequently reported in historical accounts of Northern Great Plains eating patterns. Turnips purchased at a grocery store in 2025 are quite different in taste, appearance, and texture. Yet, without the resources to grow, harvest, or forage foods for this menu, store-bought turnips were the best alternative. This same process applied to several other food items on the menu as well, notably including most of the meats (which would have been wild game versus farmed animals) as well as sweet corn (maize), which would have been a genetically different product pre-1851. Sweet corn grown by indigenous peoples of the Americas contained a recessive gene allele *sugary1* (*su1*)*,* which is within the starch biosynthesis pathway and results in higher sugar content in the kernels [56]. Agricultural practices and research have identified additional genes in the starch biosynthesis pathway, such as the recessive gene allele *shrunken2* (*sh2*), which increases the sugar content in corn kernels. Many varieties of modern “sweet corn” are referred to as “super sweet corn” because of this increased sugar content, which is 2 times that of *su1* sweet corn [57]. In addition, many of the ingredients used in the menu are much more processed today than in the 19th century or earlier. For instance, oils used in the menu are industrially refined and purified, hazelnut milk was purchased commercially, and salmon was purchased canned and/or frozen.

Some aspects of this menu were not able to be historically accurate. For instance, food is provided to participants in the intervention study in adequate amounts to meet their nutrient and energy needs. However, historically, the foods in this menu would not all have been available at the same time. Honey is now available year-round in large quantities but may not have been as readily available to indigenous communities. Several foods would have been air dried for long storage, and there would be times marked with feast and times marked with famine. This intervention menu also provided 3 regular meals per day, which participants could divide into as many eating occasions as they preferred (some participants may have consumed their food at 2 meals, some in 5 or 6 small meals throughout the day). We do not know what the eating cadence of historical indigenous peoples looked like, so we do not know if a self-determined eating pace matches traditional eating patterns. Modern cooking techniques and equipment were also used to ensure modern food safety concerns were addressed. Foods were prepared, cooked, and stored indoors using temperature-controlled appliances in lieu of an open fire or coals.

Finally, the foods in this menu were separated from the physical activity that normally would be required to obtain them. Participants were not foraging for food, farming, hunting, or performing cooking tasks, such as grinding corn, butchering animals, or drying food on homemade wooden structures. Traditionally, strenuous physical activity would have been required to access and prepare the foods in this menu.

In conclusion, although it was feasible to develop a menu that largely aligned with recommendations from the DGA while containing primarily traditional foods, there are many limitations to the knowledge we have about indigenous dietary patterns and traditional foods before the establishment of reservations. Additional research that integrates historical records, knowledge of indigenous leaders, and contemporary nutrition science methods is needed to advance the understanding of the health impacts of traditional indigenous foodways.

## Author contributions

The authors’ responsibilities were as follows – JMH, JDB, MEC: conceptualization; JMH, JDB, MEC, BF, AS, DF: methodology; JMH, JDB, MEC, BF, AS, DF: formal analysis; JDB, BF, EK, TBR: resources; JMH, JDB: writing—original draft preparation; JMH, JDB, MEC, AS, DF, DCB, JNR: writing—review and editing; and all authors: have read and agreed to the published version of the manuscript.

## Data availability statement

All data used by this study can be found in the cited references.

## Funding

This work was supported by the USDA Agricultural Research Service project grant #3062-10700-003-000D (JMH, JNR) and an ARS Administrator-funded Research Associate Award (JNR).

## Conflict of interest

This research was led by the USDA-ARS Grand Forks Human Nutrition Research Center (GFHNRC), which includes Nutrition and Native American Health as one of its research focuses. Native American/Indigenous Health remains an understudied aspect of nutrition science and research. JMH, JDB, MEC, BF, DF, AS, DCB, and JNR were employees of the GFHNRC while this research was being conducted and are residents of the Grand Forks community. This work was conducted in partnership with United Tribes Technical College, a tribal college in Bismarck, ND, United States. EK is a staff member of UTTC, and TBR is a staff member at Sitting Bull College, a tribal college on the Standing Rock Reservation in ND, United States.
