# Supplementary material for: Creating a Nutrient-Dense Menu Using Foods Consumed by Native Communities in the Northern Great Plains Prior to 1851 for Use in Dietary Intervention Trial
Source: Curr Dev Nutr. 2025 Aug 5;9(9):107522. doi: 10.1016/j.cdnut.2025.107522 (PMC12512147; doi:10.1016/j.cdnut.2025.107522)

# Supplementary Materials for Hess et al. Creating a Nutrient-Dense Menu Using Foods Consumed by Native Communities in the Northern Great Plains Prior to 1851 for Use in Dietary Intervention Trial

## Supplementary Table 1. References for Recipes used in Healthy Indigenous Menu

| Day/Meal | Recipe Name | Source |
| --- | --- | --- |
| Day 1/Breakfast (D1B) | Cornmeal Mush | The Sioux Chef’s Indigenous Kitchen, p.59 |
| Day 1/Lunch (D1L) | Aronia Berry Juice | n/a |
| D1L | 3 Sisters Stew | New York Times |
| D1L | Wild Green Salad with Walnuts | Cherokee Tribal Food Distribution Program (Lois Ellen Frank) |
| D1L | Wild Rice Patty | Indigi Kitchen |
| Day 1/Supper (D1S) | Berry Fruit Salad | Dietary Core |
| D1S | Green Beans, Hazelnuts & Dried Cranberries | Northwest Kidney Centers |
| D2B | Sweet Potato Breakfast Scramble | Indigi Kitchen |
| D2B | Amaranth with Berries | The Sioux Chef’s Indigenous Kitchen, p.195 |
| D2L | Simple Corn Cakes | The Sioux Chef’s Indigenous Kitchen (included in the bison tacos), p.122 |
| D2L | Mashed Cedar Braised Beans | The Sioux Chef’s Indigenous Kitchen, p. 36-37 |
| D2L | Jicama Radish Salad | American Indian Health and Diet Project |
| D2L | Indigenous Bison Tacos | The Sioux Chef’s Indigenous Kitchen, p. 122 |
| D2L | Unstuffed Bell Peppers | American Indian Health and Diet Project |
| D2S | Maple Sage Roasted Vegetables | The Sioux Chef’s Indigenous Kitchen, p. 46 |
| D3B | Wild Rice Pudding Cake | The Sioux Chef’s Indigenous Kitchen, p. 145 |
| D3B | Wojape | The Sioux Chef’s Indigenous Kitchen, p. 173 |
| D3L | Salmon Cornmeal Cakes | Indigi Kitchen |
| D3L | Summer Squash with Scallions, Chile, Parsley | Real Simple (Ananda Eidelstein) |
| D3S | Venison Burgers | American Indian Health and Diet Project |
| D3S | Wild Green with Flowers Salad with Dressing | Paula Giese |
| D3S | Zucchini Canoes | Nicolette Pace (Farmers Market Friday) |
| D4B | Chokeberry Juice | Developed by Dietary |
| D4B | Scrambled Eggs | Developed by Dietary |
| D4B | Roasted Purple Potato | Developed by Dietary |
| D4L | Bison Chili | Beautiful Badlands (Mary Tastad) |
| D4L | No Mayo Coleslaw | Camila’s |
| D4L | Cornbread | Jiffy |
| D4S | Aronia Berry Juice | n/a |
| D4S | Pan Seared Walleye | Cherokee Tribal Food Distribution |
| D4S | Three Sisters Salad with Greens | Healing With Plants |
| D4S | Pesto-tossed spaghetti squash topped with 3 sisters salad | n/a |
| D5B | Potawatomi Berry Rice | Indigi Kitchen |
| D5L | Manoomin Elk Meatballs | Well For Culture |
| D5L | Succotash | A couple Cooks (Sonja Overhiser) |
| D5L | Simple Corn Cakes | The Sioux Chef’s Indigenous Kitchen, p. 122 |
| D5S | Roast Turkey with Crushed Cranberry Compote | Sur La Table Chef Jordan Carfagno (National Museum of American History) |
| D5S | 3 Sisters Mash | The Sioux Chef’s Indigenous Kitchen, p. 43 |
| D5S | Pinon Cakes | Healthy Traditions: Recipes of Our Ancestors (Janice Goodwin and Judy Hall) |

## Supplementary Table 2: Bison Chili Ingredients

| **BISON CHILI** |
| --- |
| **INGREDIENTS** |
| Ground Bison |
| Yellow Onion, diced |
| Pinto Beans, no salt added, rinsed, drained |
| Tomatoes, diced, no salt added, basil/garlic/oregano added |
| Water |
| Chili powder |
| Cumin, ground |
| Salt |
| Black pepper, ground |

## Supplementary Table 3: Cornmeal Mush Ingredients

| **CORNMEAL MUSH / POLENTA** |
| --- |
| **INGREDIENTS** |
| Cornmeal, yellow |
| Water |
| Salt |

## Supplementary Table 4: Hard Cooked Eggs Ingredients

| **HARD COOKED EGGS** |
| --- |
| **INGREDIENTS** |
| Eggs (large) |

## Supplementary Table 5: Aronia Berry Juice Drink Ingredients

| **ARONIA BERRY JUICE DRINK** |
| --- |
| **INGREDIENTS** |
| Tap Water (room temperature) |
| Aronia Powder (1Tbsp = 10gm) |
| Maple Syrup |

## Supplementary Table 6: Three Sister’s Stew Ingredients

| **THREE SISTER’S STEW** |
| --- |
| **INGREDIENTS** |
| Garlic Paste |
| Yellow Onion, diced |
| Green Bell Pepper |
| Tomatoes, canned, diced, basil, garlic, and oregano, low sodium |
| Black Beans, canned, low sodium; drained and rinsed |
| Corn, frozen |
| Butternut Squash, frozen |
| Cumin, ground |
| Oregano |
| Salt |
| Black Pepper, ground |
| Chicken Broth, carton, low sodium |

## Supplementary Table 7: Venison Tips Ingredients

| **VENSION* TIPS (for 3 Sister’s Stew)** |
| --- |
| **INGREDIENTS** |
| Venison (Tips) |
| Broth |

## Supplementary Table 8: Ground Bison Ingredients

| **Ground BISON (for 3 Sister’s Stew)** |
| --- |
| **INGREDIENTS** |
| Ground Bison, raw |

## Supplementary Table 9: Mixed Green Salad Ingredients

| **MIXED GREENS SALAD** |  |
| --- | --- |
| **INGREDIENTS** |  |
| Mixed salad greens, raw |  |
| Raisins |  |
| Walnuts, chopped, toasted |  |
| Bragg Salad Dressing |  |
| Supplementary Table 10: Wild Rice Flatbread Ingredients | |
| **WILD RICE FLATBREAD** | |
| **INGREDIENTS** | |
| Wild Rice, cooked | |
| Egg White, raw | |
| Water | |
| Chia Seeds | |
| Garlic Powder | |
| Red Pepper Flakes | |

## Supplementary Table 11: Wild Rice & Salmon Ingredients

| **WILD RICE & SALMON** |
| --- |
| **INGREDIENTS** |
| Wild Rice, cooked |
| Salmon, raw |
| Lemon Juice |
| Lemon Pepper |

## Supplementary Table 12: Seasoned Green Beans & Walnuts Ingredients

| **SEASONED GREEN BEANS & WALNUTS** |
| --- |
| **INGREDIENTS** |
| Green Beans, whole, frozen |
| Sunflower oil |
| Juniper, ground |
| Walnuts, chopped, toasted |

## Supplementary Table 13: Berry Fruit Salad Ingredients

| **BERRY FRUIT SALAD** |
| --- |
| **INGREDIENTS** |
| Mixed Berries (blueberry, strawberry, raspberry, blackberry), frozen |
| DRESSING |
| Honey |
| Lime Juice |

## Supplementary Table 14: Sweet Potato Breakfast Scramble Ingredients

| **SWEET POTATO BREAKFAST SCRAMBLE** |  |  |
| --- | --- | --- |
| **INGREDIENTS** |  |  |
| Sweet Potato, peeled, diced |  |  |
| Green Bell Pepper, diced |  |  |
| Green Onion, diced |  |  |
| Eggs, large, raw |  |  |
| Supplementary Table 15: Amaranth Ingredients |  |  |
| **AMARANTH** (w/ Wojape [wild berries]) |  |  |
| **INGREDIENTS** |  |  |
| Amaranth |  |  |
| Water |  |  |
| Salt |  |  |
| Supplementary Table 16: Wojape- Mixed Berry Sauce Ingredients | |  |
| **WOJAPE - MIXED BERRY SAUCE** | |  |
| **INGREDIENTS** | |  |
| Frozen Berry Mix (blueberry, strawberry, raspberry, blackberry) | |  |
| Supplementary Table 17: Ground Bison Taco Meat Ingredients | |  |
| **Ground BISON Taco Meat** | |  |
| **INGREDIENTS** | |  |
| Ground Bison, raw | |  |
| Taco Seasoning, Ortega | |  |
| Water | |  |
| Supplementary Table 18: Roasted Onion & Peppers Ingredients | |  |
| **ROASTED ONION & PEPPERS** | |  |
| **INGREDIENTS** | |  |
| Red Peppers, cut into strips | |  |
| Green Peppers, cut into strips | |  |
| Yellow Onion, cut into strips | |  |
| Supplementary Table 19: Radish Salsa Ingredients | | |
| **RADISH SALSA** | | |
| **INGREDIENTS** | | |
| Radishes, stems removed, washed | | |
| Garlic Paste | | |
| Jalapeno, ribs and seeds removed | | |
| Lemon juice | | |
| Cilantro | | |
| Salt | | |
| Pepper | | |

## Supplementary Table 20: Bison Hash Ingredients

| **Bison Hash** |
| --- |
| **INGREDIENTS** |
| Ground Bison, raw |
| Salt |
| Pepper |
| Garlic powder |
| Yellow onion |
| Juniper, ground (1 tsp.) |
| Cumin |
| Water |
| Sweet Potato, raw, diced |
| Turnip, raw, diced |
| Winter squash, butternut, frozen, diced |
| Wild rice, cooked |

## Supplementary Table 21: Corn Muffin Ingredients

| **CORN MUFFIN** |
| --- |
| **INGREDIENTS** |
| Cornbread mix, Bob’s Red Mill |
| Hazelnut ‘Milk’ Beverage |
| Egg |
| Sunflower Oil |

## Supplementary Table 22: Sunflower Cookies Ingredients

| **Sunflower Cookies** |
| --- |
| **INGREDIENTS** |
| SunButter, natural |
| Maple Syrup |
| Salt |
| Cornmeal |
| Sunflower seeds, hulled, kernels |
| Cornmeal |

## Supplementary Table 23: Wild Rice Porridge Ingredients

| **WILD RICE PORRIDGE** |
| --- |
| **INGREDIENTS** |
| Wild Rice, salted, cooked |
| Hazelnut (milk) Beverage |

## Supplementary Table 24: Salmon Patty Ingredients

| **SALMON PATTY** |
| --- |
| **INGREDIENTS** |
| Salmon (skinless, boneless), canned, drained |
| Egg, beaten, large, raw |
| Water |
| Lemon Juice |
| Dijon Mustard |
| Dill, dried |
| Garlic Powder |
| Lemon Pepper |
| Masa Flour |

## Supplementary Table 25: Lemon Dill Dipping Sauce Ingredients

| **LEMON DILL DIPPING SAUCE** |
| --- |
| **INGREDIENTS** |
| Avocado Mayonnaise |
| Dill, dried |
| Lemon Juice |
|  |

## Supplementary Table 26: Braised Beans Ingredients

| **BRAISED BEANS** |
| --- |
| **INGREDIENTS** |
| Pinto Beans, canned (15.5oz), low sodium, rinsed, drained |
| Yellow Onion, diced |
| Garlic paste |
| Chicken Broth, carton, low sodium |

## Supplementary Table 27: Summer Squash Salad Ingredients

| **SUMMER SQUASH SALAD** |
| --- |
| **INGREDIENTS** |
| Zucchini |
| Summer Squash |
| Parsley |
| Green Onion |
| DRESSING |
| Sunflower Oil |
| Apple Cider Vinegar |
| Salt |
| Juniper |

## Supplementary Table 28: Elk Burger Ingredients

| **ELK BURGER** |
| --- |
| **INGREDIENTS** |
| Elk burger patty |
| Salt |
| Pepper |

## Supplementary Table 29: Wild Greens Salad Ingredients

| **WILD GREENS SALAD** |
| --- |
| **INGREDIENTS** |
| Mixed salad greens, raw |
| Black Beans, canned, low sodium |
| Bragg Salad Dressing |

## Supplementary Table 30: Stewed Zucchini Ingredients

| **STEWED ZUCCHINI** |
| --- |
| **INGREDIENTS** |
| Sunflower Oil |
| Yellow onion, diced |
| Zucchini, diced |
| Tomatoes with basil, garlic, and oregano, canned, diced, NSA (14.5 oz) |
| Wild Rice |

## Supplementary Table 31: Scrambled Eggs Ingredients

| **SCRAMBLED EGGS** |
| --- |
| **INGREDIENTS** |
| Avocado Oil Spray |
| Eggs, large, raw |
| Mushrooms, sliced |
| Yellow Onion, diced |
| Spinach, raw |

## Supplementary Table 32: Purple Roasted Potatoes Ingredients

| **PURPLE ROASTED POTATOES** |
| --- |
| **INGREDIENTS** |
| Avocado Oil Spray |
| Purple Potatoes, raw, diced |
| Mrs. Dash |
| Sunflower Oil |

## Supplementary Table 33: No Mayo Coleslaw Ingredients

| **NO MAYO COLESLAW** |
| --- |
| **INGREDIENTS** |
| Coleslaw, bagged mix |
| Green Onion, diced |
| DRESSING |
| Apple Cider Vinegar |
| Maple Syrup |
| Dijon mustard |
| Celery Seeds |

## Supplementary Table 34: Three Sister’s Salad Ingredients

| **THREE SISTER’S SALAD** |
| --- |
| **INGREDIENTS** |
| Black Beans. Canned, rinsed and drained |
| Corn, frozen |
| Zucchini, includes skin, raw, chopped |
| Yellow Squash, includes skin, raw, chopped |
| Yellow Onion, diced |
| DRESSING |
| Maple Syrup |
| Apple Cider Vinegar |
| Sunflower Oil |

## Supplementary Table 35: Spaghetti Squash with Pesto Ingredients

| **SPAGHETTI SQUASH w/ PESTO** |
| --- |
| **INGREDIENTS** |
| Spaghetti squash, roasted |
| Pesto |

## Supplementary Table 36: Potawatomi Berry Rice Ingredients

| **POTAWATOMI BERRY RICE** |
| --- |
| **INGREDIENTS** |
| Wild rice, cooked |
| Mixed berries (blueberry, strawberry, raspberry, blackberry), frozen |
| Maple syrup |
| Cinnamon |
| Walnuts |

## Supplementary Table 37: Manoomin (Wild Rice) Elk Meatballs Ingredients

| **MANOOMIN (wild rice) ELK MEATBALLS** |
| --- |
| **INGREDIENTS** |
| Elk, ground, raw |
| Wild rice, cooked, salted |
| Egg, raw |
| Yellow onion |
| Garlic powder |
| Black pepper |
| Salt |
| Avocado oil spray |

## Supplementary Table 38: Succotash Ingredients

| **SUCCOTASH** |
| --- |
| **INGREDIENTS** |
| Sunflower Oil |
| Yellow Onion, minced |
| Corn, frozen |
| Lima Bean |
| Red Bell Pepper, finely diced |
| Grape Tomato, halved |
| Garlic powder |
| Smoked Paprika |
| Sage, ground |
| Salt |
| Juniper, ground |

## Supplementary Table 39: Gooseberry Compote Ingredients

| **GOOSEBERRY COMPOTE** |
| --- |
| **INGREDIENTS** |
| Gooseberries, canned, with liquid |
| Cranberries, dried, sweetened with apple juice |

## Supplementary Table 40: Caramelized Seed Mix Ingredients

| **CARAMELIZED SEED MIX** |
| --- |
| **INGREDIENTS** |
| Pepitas |
| Sunflower seeds, hulled, kernels |
| Walnuts, chopped, toasted |
| Maple Sugar |

# Supplementary Table 41. Energy contributed by non-traditional Indigenous foods to the Healthy Indigenous Menu

| **Study 157 Foods_20%** | |
| --- | --- |
| **DAY 1** | **KCAL** |
| *BREAKFAST* |  |
| *LUNCH* |  |
| Cumin | .13 |
| Garlic | 3.8 |
| Braggs dressing | 60 |
| Red Pepper Flakes | 0.2 |
| Garlic | 1.2 |
| Chia Seeds | 6.3 |
| *SUPPER* |  |
| Lemon juice | 1.7 |
| Lemon pepper | 5 |
| Lime juice | 2.1 |
|  | **80.43** |
| **DAY 2** |  |
| *BREAKFAST* |  |
| *LUNCH* |  |
| Taco seasoning | 32.2 |
| Cilantro | 0.1 |
| Lemon Juice | 0.6 |
| Jalapeno | 1 |
| Garlic | 1.3 |
| *SUPPER* |  |
| Garlic | 2 |
| Cumin | 3.8 |
|  | **41** |
| **DAY 3** |  |
| *BREAKFAST* |  |
| *LUNCH* |  |
| Lemon dill dipping sauce | 188 |
| Parsley | 3.6 |
| *SUPPER* |  |
| Braggs dressing | 72 |
| Red Pepper Flakes | 0.3 |
| Garlic | 1.6 |
| Chia Seeds | 8.4 |
|  | **273.9** |
| **DAY 4** |  |
| *BREAKFAST* |  |
| Dash Seasoning | 7 |
| *LUNCH* |  |
| Cider Vinegar | 5 |
| Dijon mustard | 9 |
| Celery seed | 2 |
| *SUPPER* |  |
| Cider Vinegar | 2 |
| Pesto sauce | 145 |
|  | **170** |
| **DAY 5** |  |
| *BREAKFAST* |  |
| Cinnamon | 2 |
| *LUNCH* |  |
| Smoked Paprika | 0.5 |
| Garlic | 1.7 |
| *SUPPER* |  |
| Compote | 73 |
| Red Pepper Flakes | 0.2 |
| Garlic | 1.2 |
| Chia Seeds | 6.3 |
|  | **84.9** |

650.23 kcal from non-traditional foods = 24% of total energy

# Supplementary Figures: Menu Photos

## Supplementary Figure 1: Day 1 Breakfast


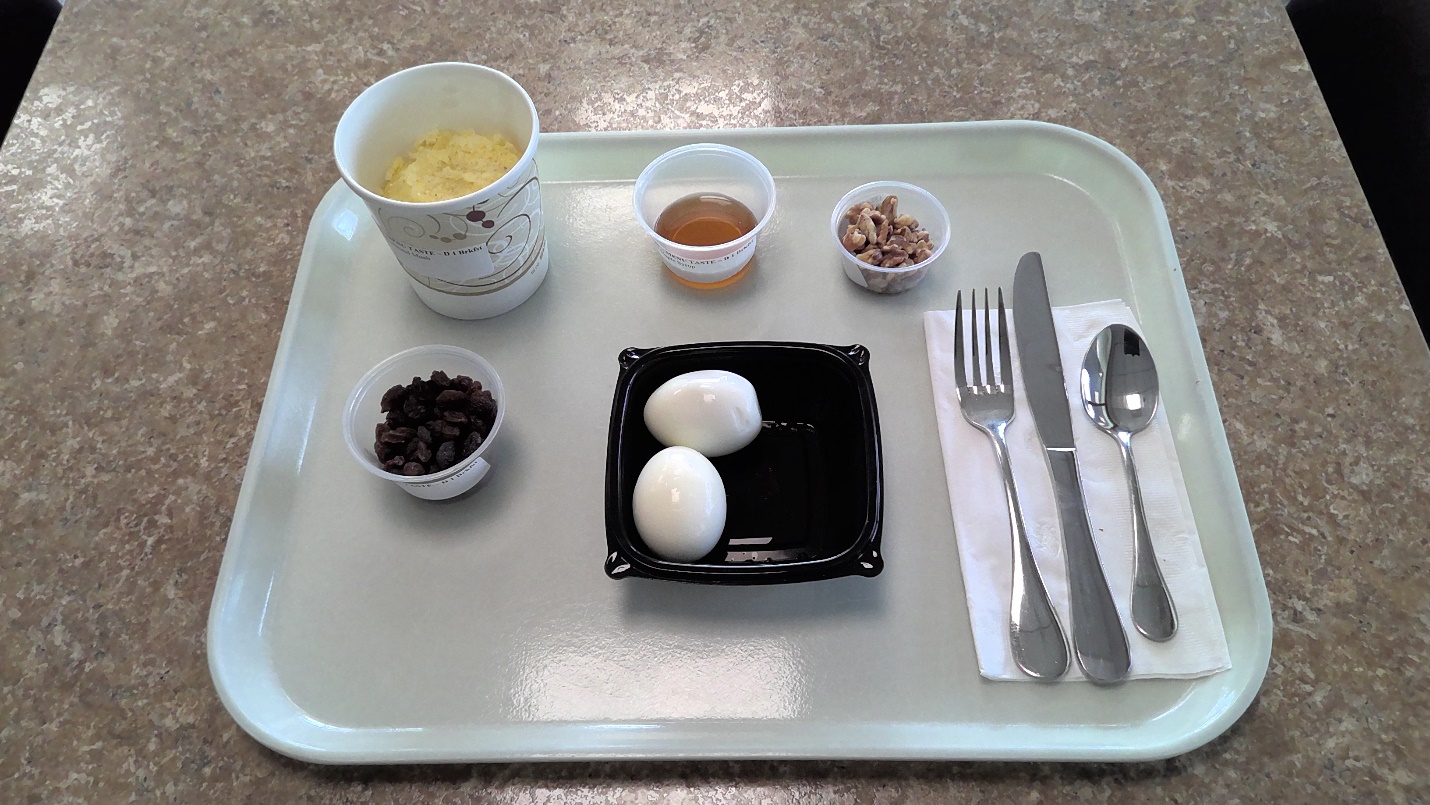


## Supplementary Figure 2: Day 1 Lunch


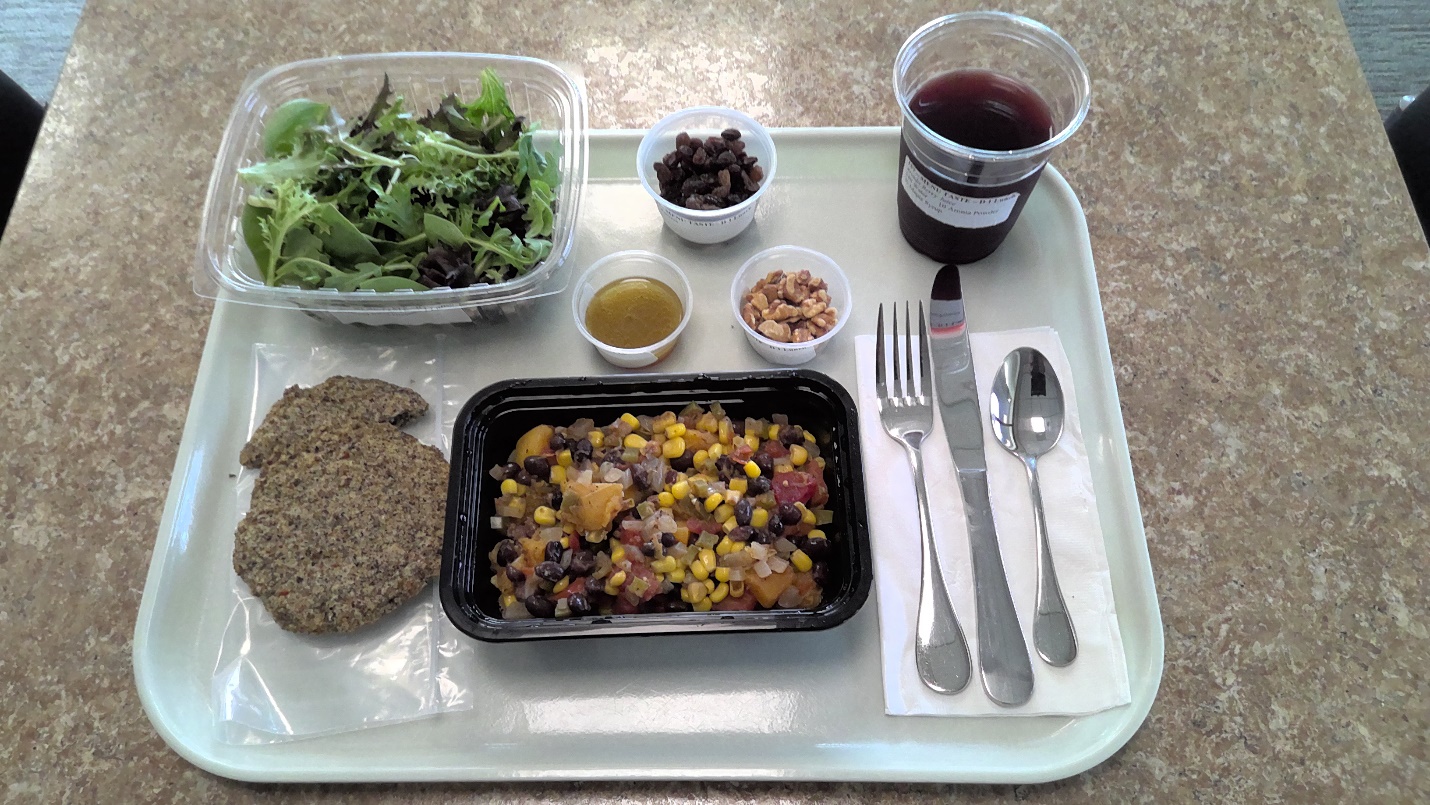


## Supplementary Figure 3: Day 1 Supper


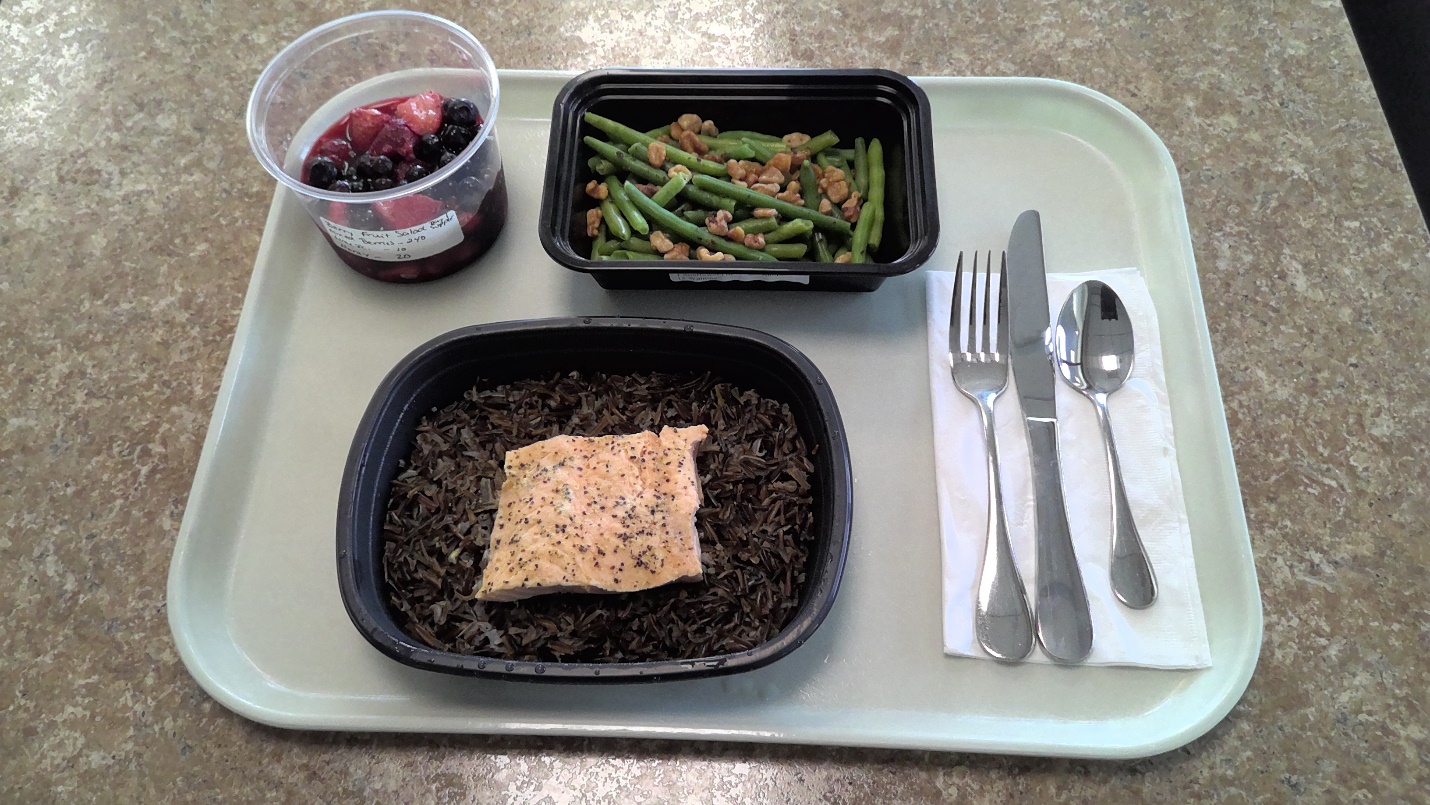


## Supplementary Figure 4: Day 2 Breakfast


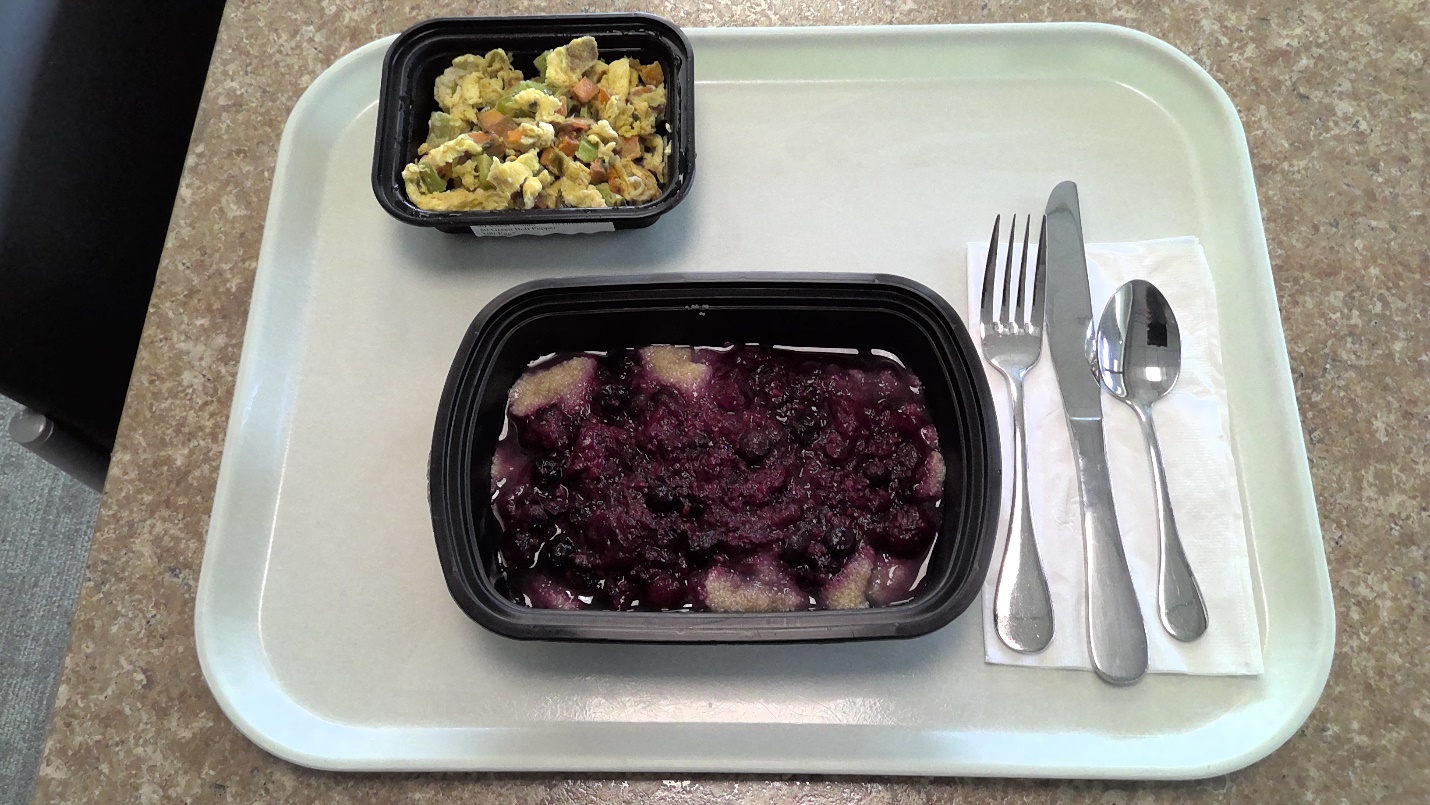


## Supplementary Figure 5: Day 2 Lunch


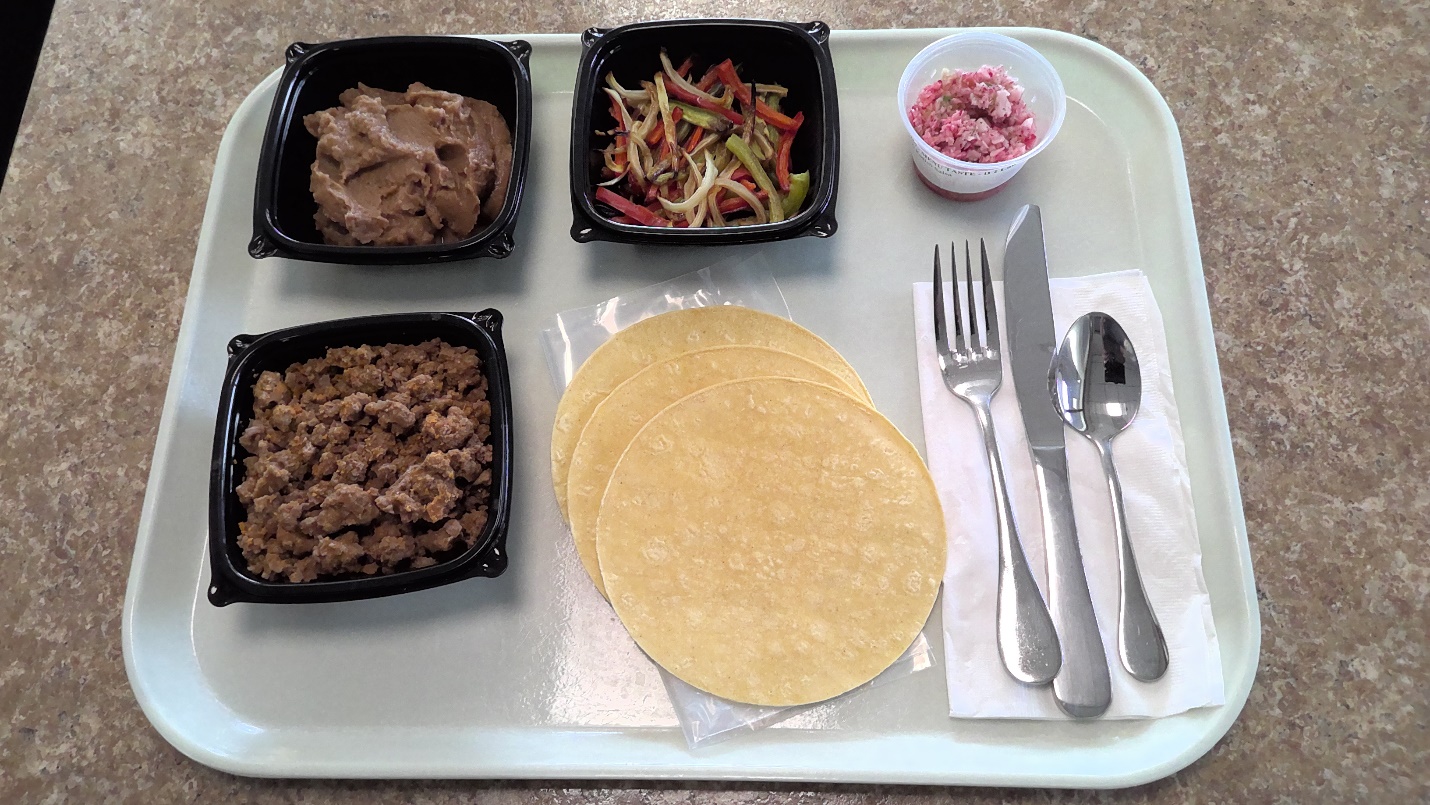


## Supplementary Figure 6: Day 2 Supper


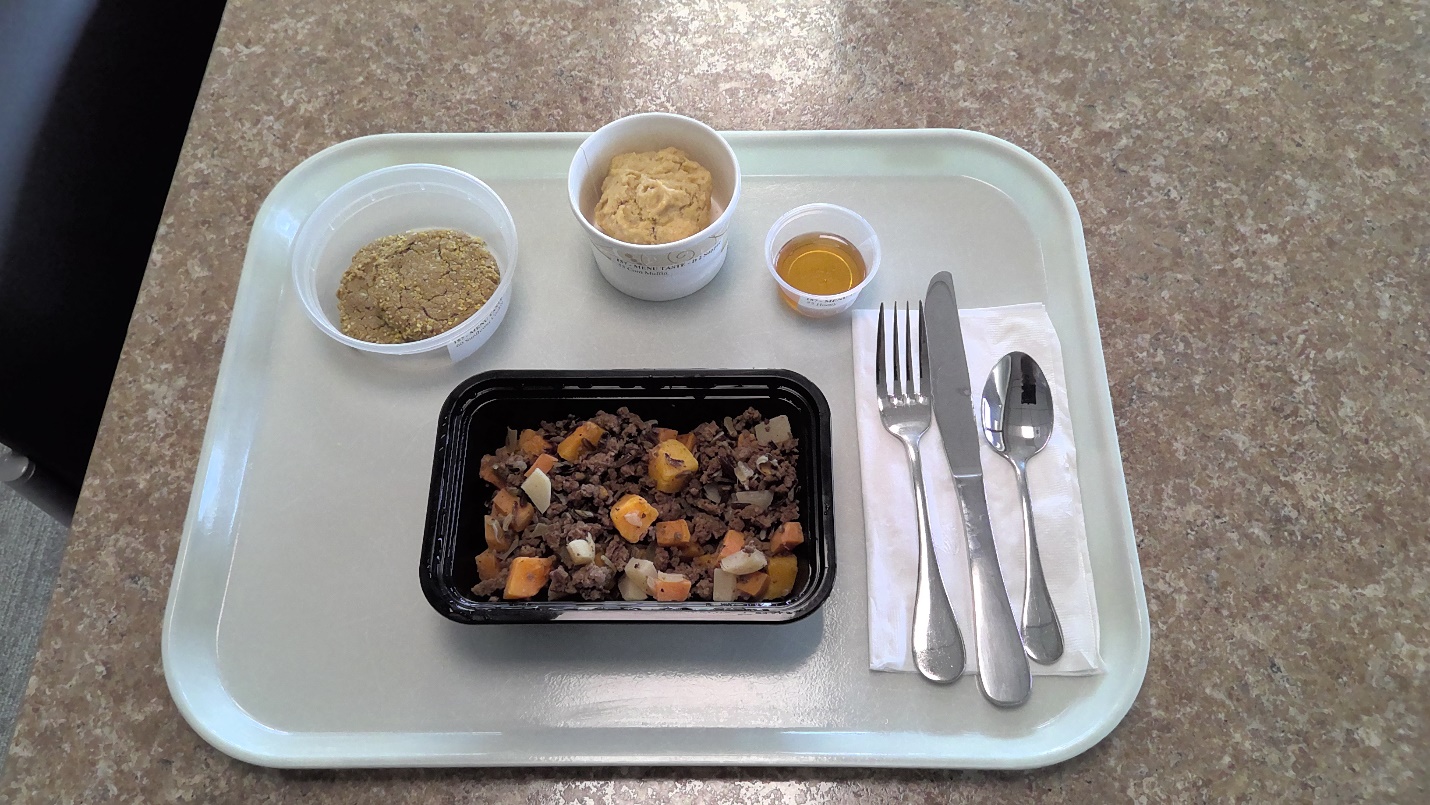


## Supplementary Figure 7: Day 3 Breakfast


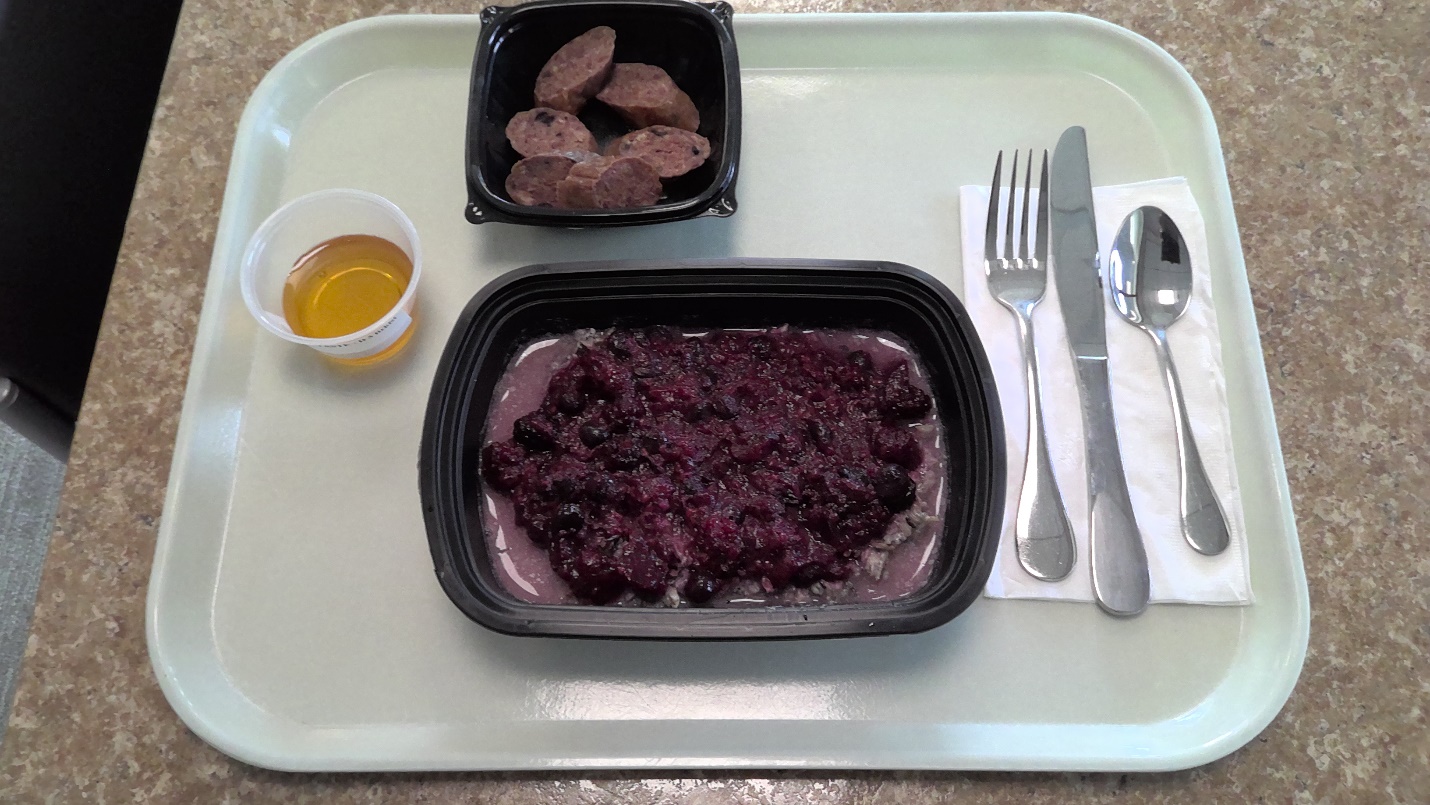


## Supplementary Figure 8: Day 3 Lunch


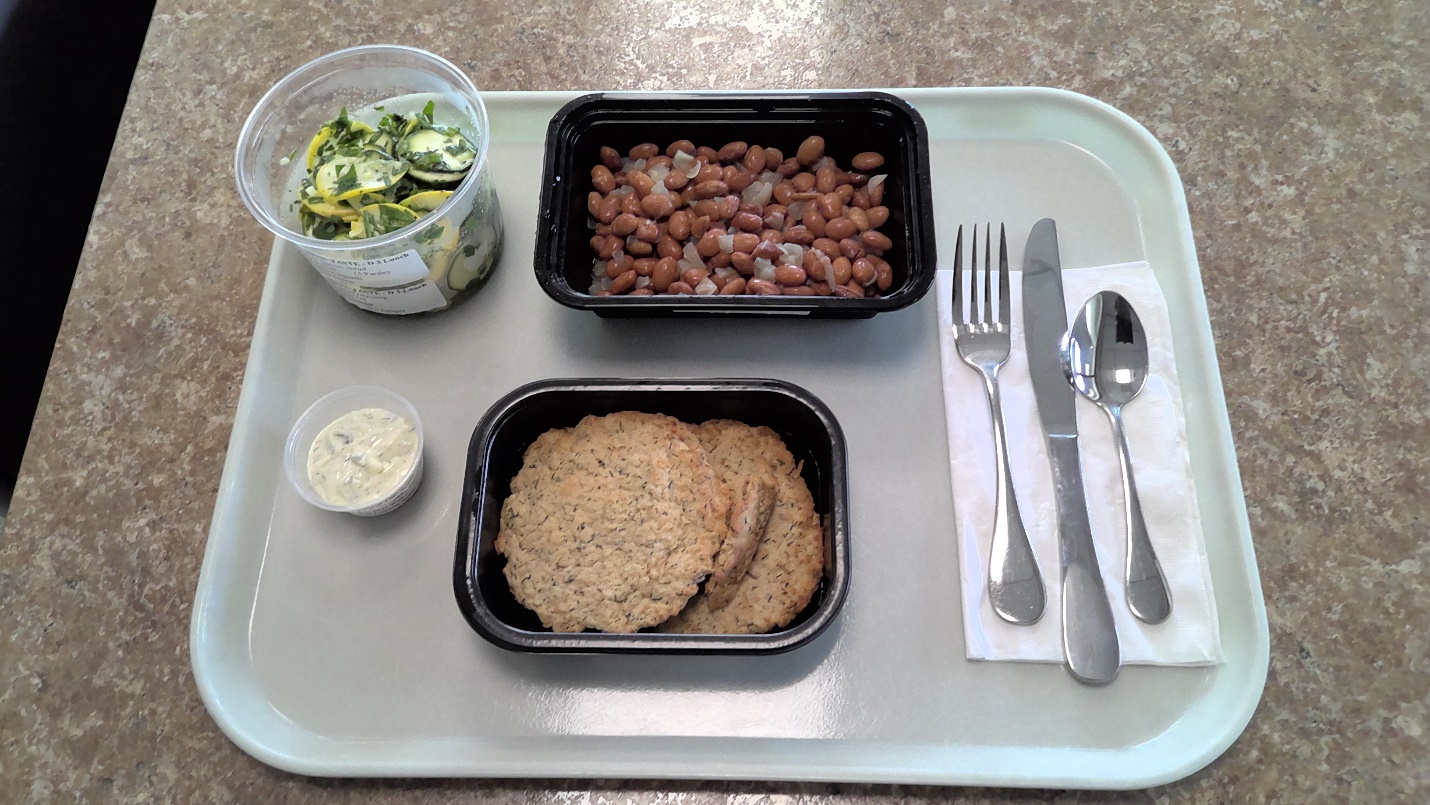


## Supplementary Figure 9: Day 3 Supper


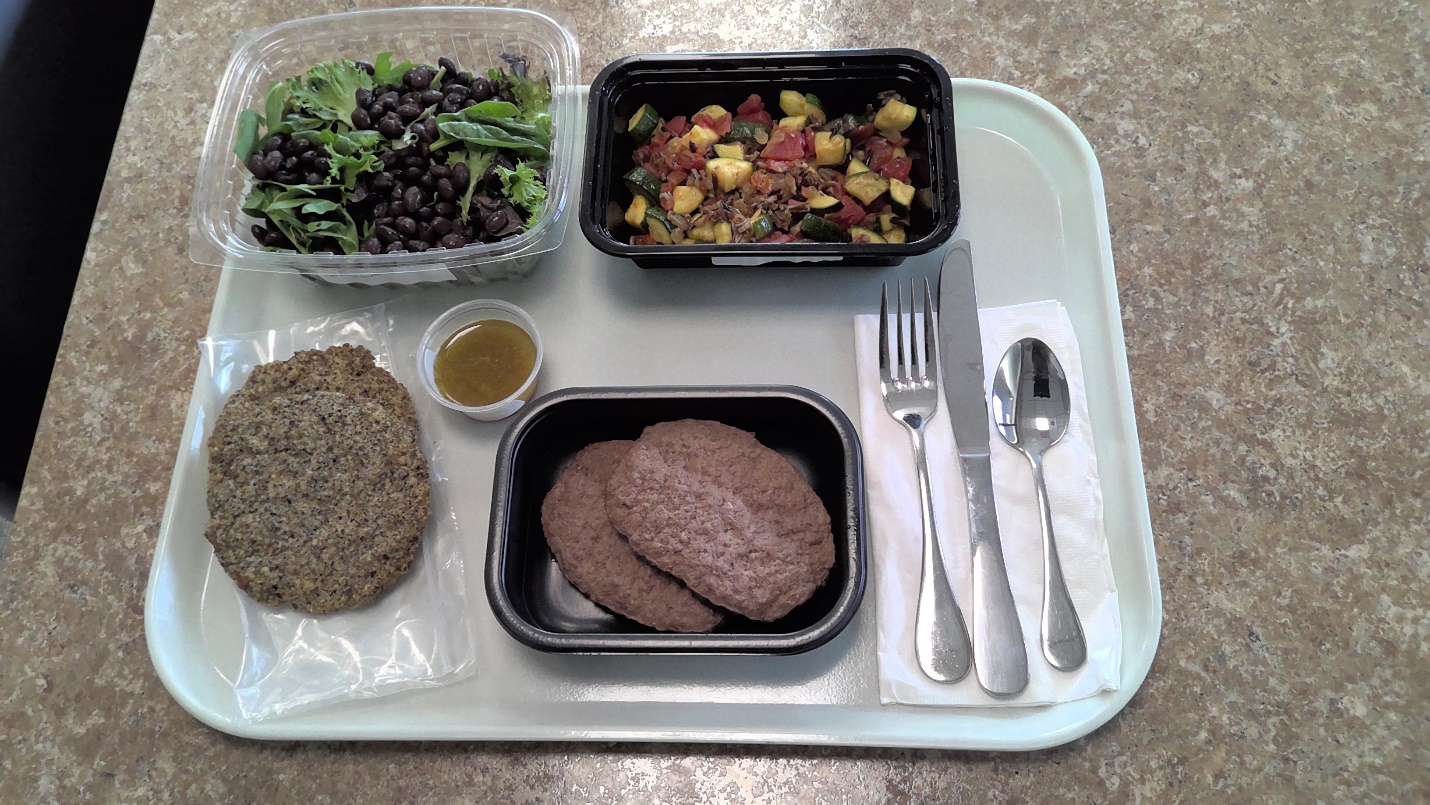


## Supplementary Figure 10: Day 4 Breakfast


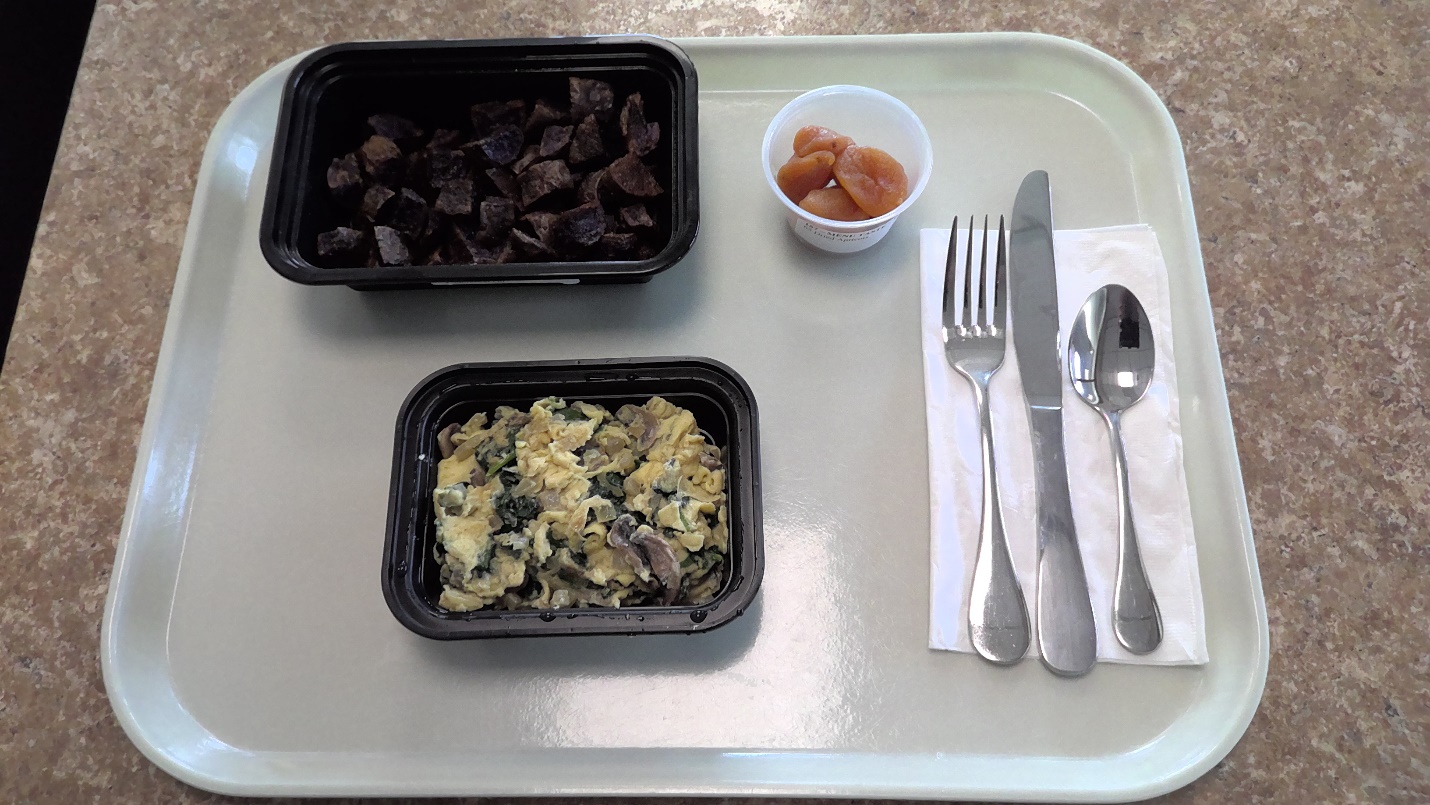


## Supplementary Figure 11: Day 4 Lunch


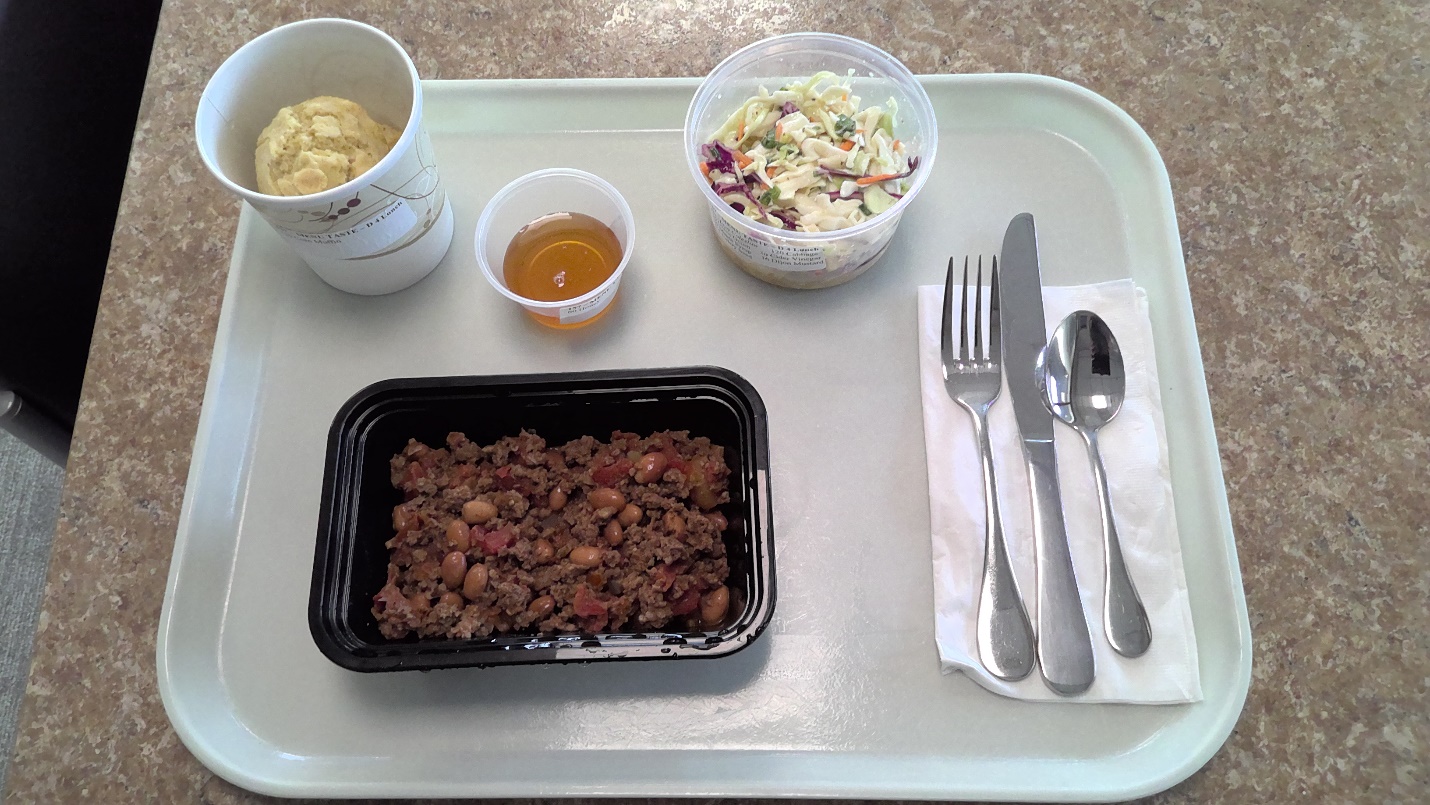


## Supplementary Figure 12: Day 4 Supper


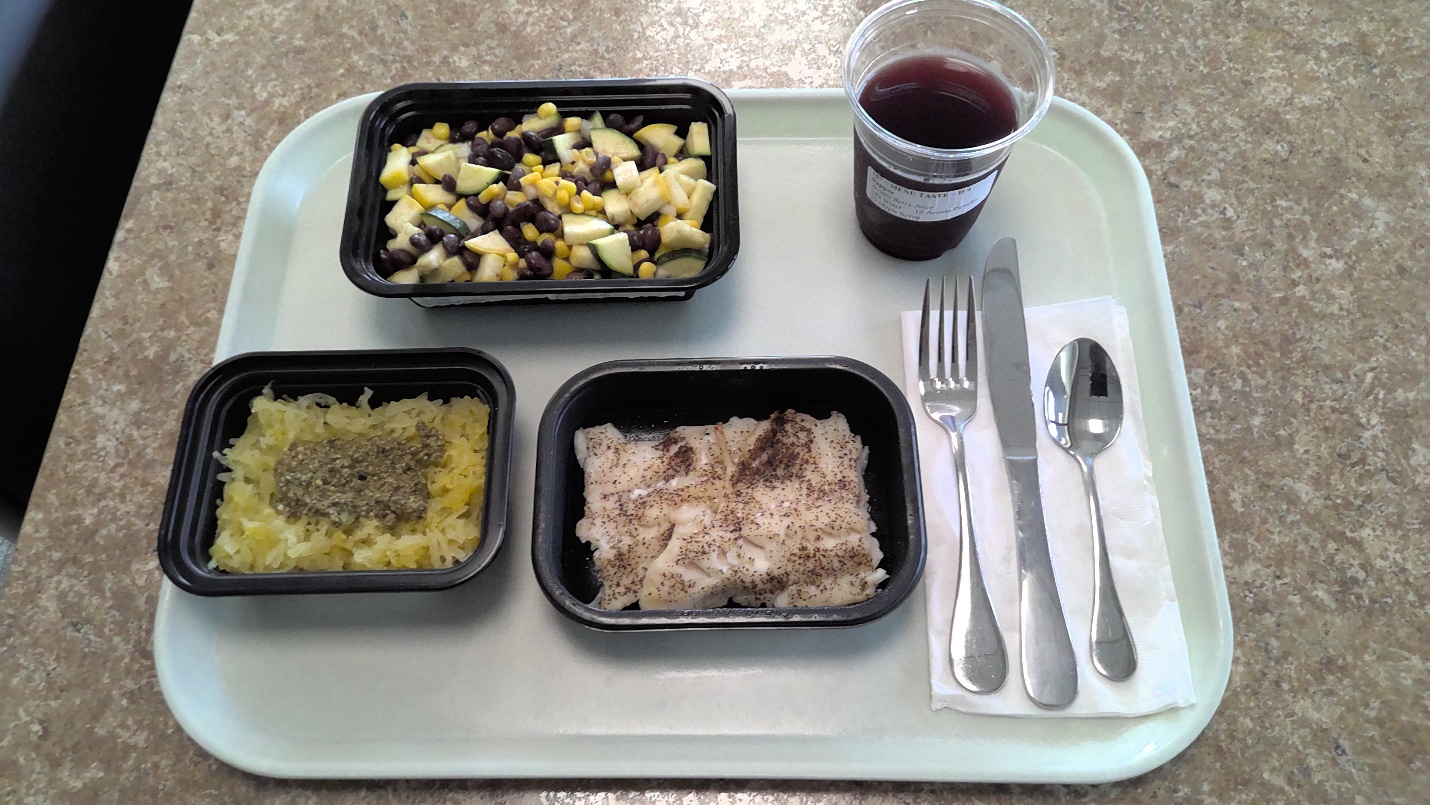


## Supplementary Figure 13: Day 5 Breakfast


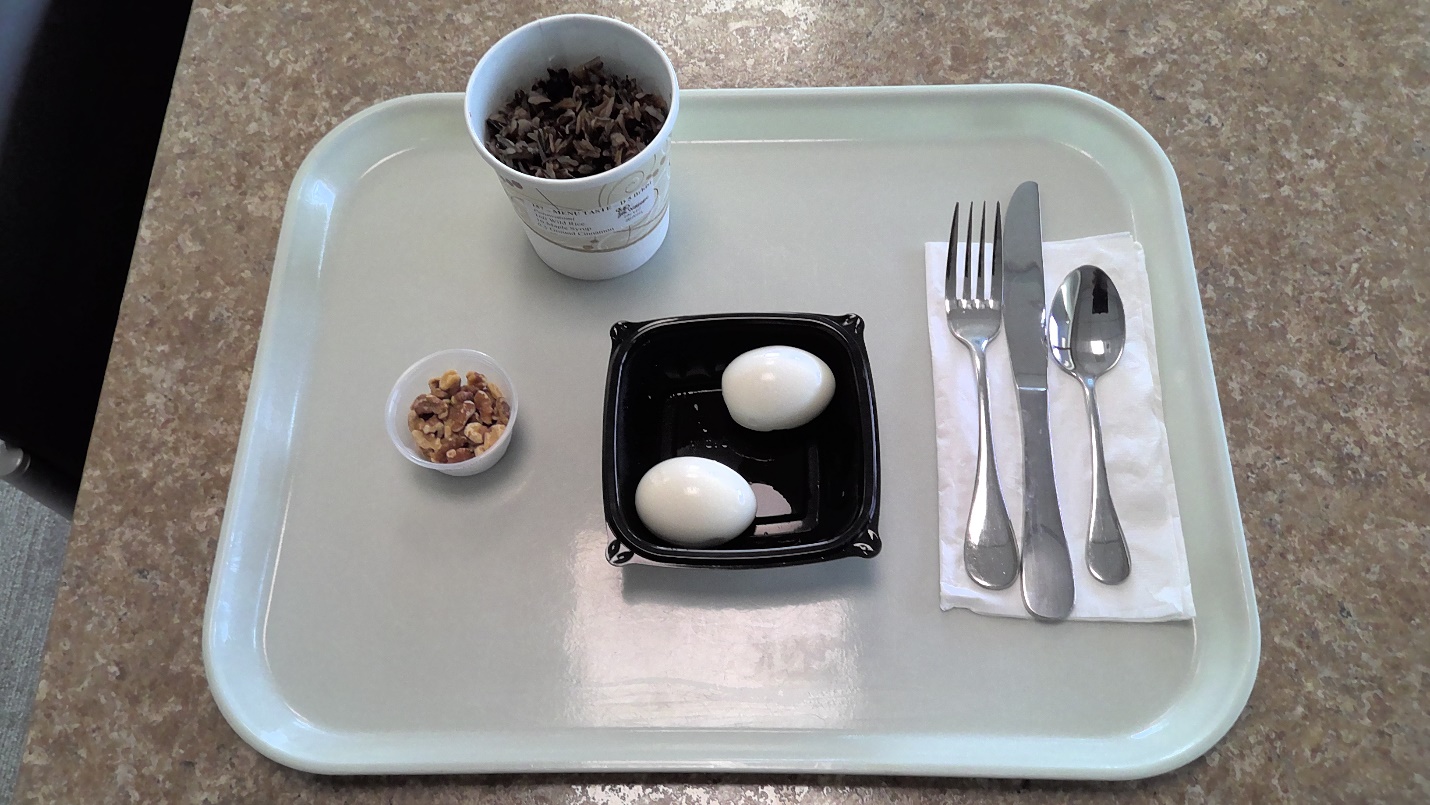


## Supplementary Figure 14: Day 5 Lunch


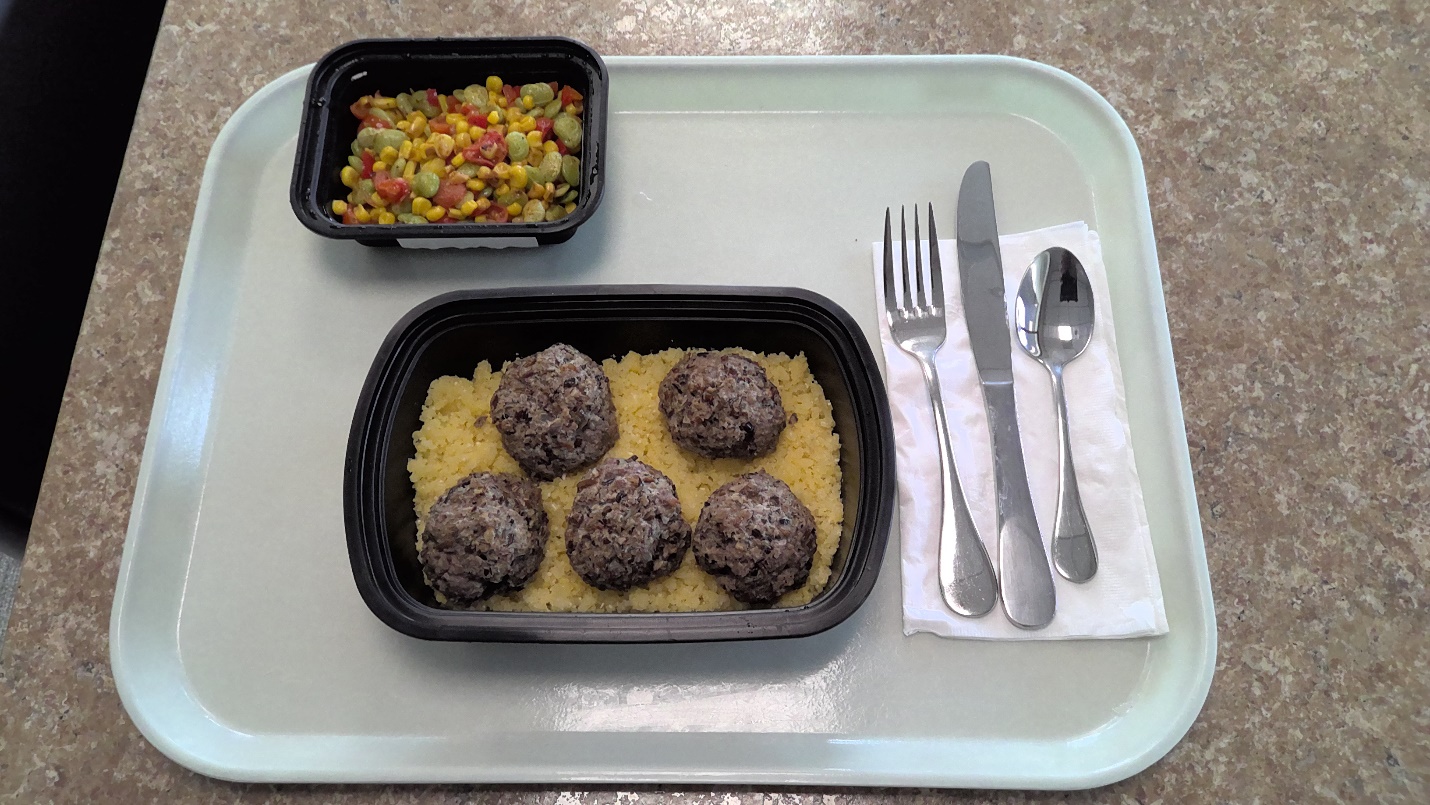


## Supplementary Figure 15: Day 5 Supper


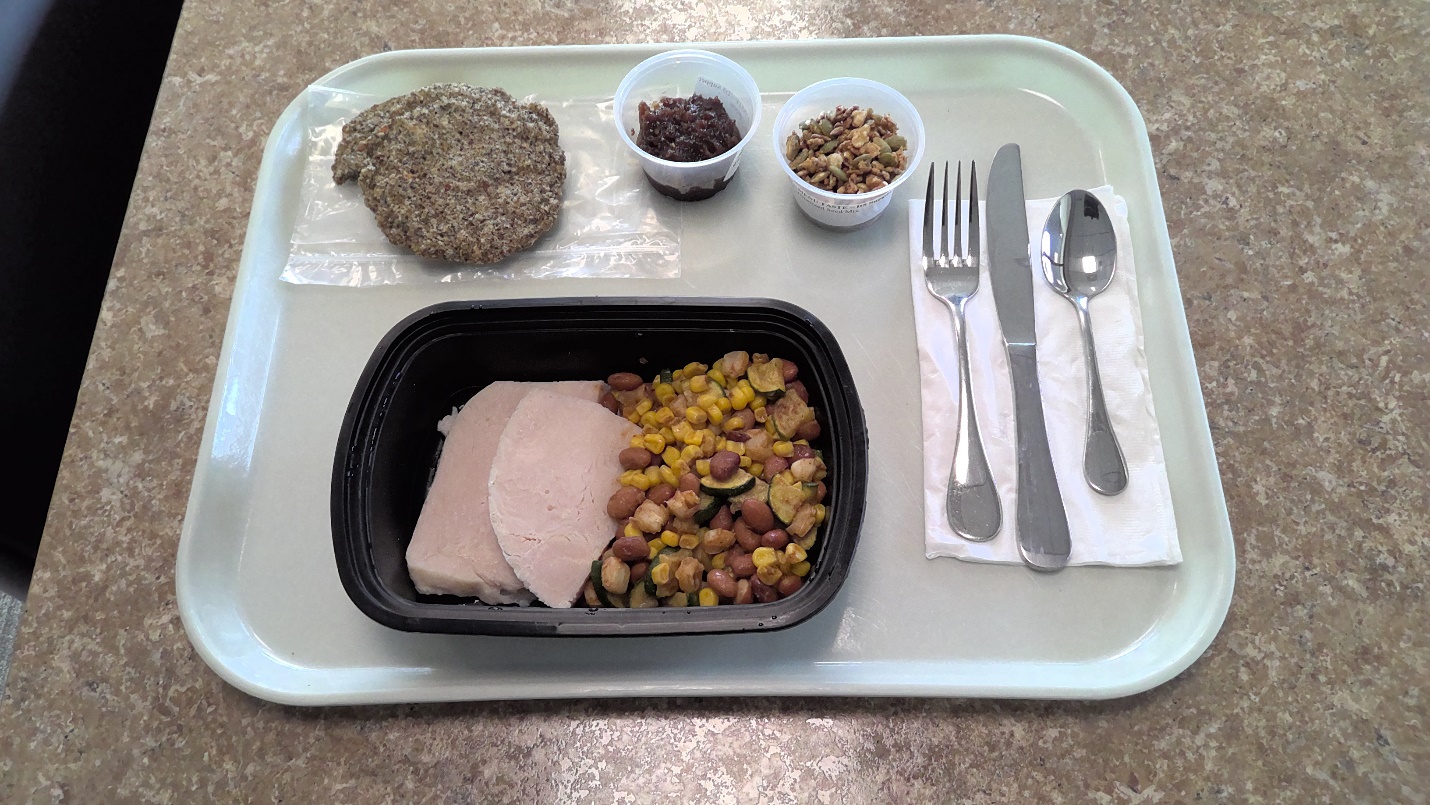

Supplement: multimedia component 1 [file mmc1.docx]
